# Supplementary material for: Variants in the β-globin locus are associated with pneumonia in African American children
Source: HGG Adv. 2024 Oct 22;6(1):100374. doi: 10.1016/j.xhgg.2024.100374 (PMC11664401; doi:10.1016/j.xhgg.2024.100374)
Supplement: Document S1. Figures S1–S9 and Table S1–S9 [file mmc1.pdf]

HGGA, Volume *6*

Supplemental information

Variants in the β-globin locus are associated with pneumonia in African American children

Nadine L.N. Halligan, Sarah C. Hanks, Karen Matsuo, Taylor Martins, Sebastian Zöllner, Michael W. Quasney, Laura J. Scott, and Mary K. Dahmer

Supplemental Information Table of Contents

Table S1. Number of Variants used for Imputation and Obtained from Imputation of Pneumonia Case/control Dataset and Imputation Reference Panel.

Figure S1. Genotype Principal Component Analysis (PCA) from PLINK and Genetic Ancestry PCA from LASER/TRACE of Total Sample before and after Removing Samples with PC1 < -0.025.

Figure S2. Genetic Ancestry Principal Components from LASER/TRACE Analysis in the Total Sample and Human Genome Diversity Project Samples.

Figure S3. Observed Imputation Quality of InPSYght Genotypes.

Table S2. Average Effective Sample Size Adjusted for Imputation.

Table S3. Estimated and Observed Imputation Quality of β-globin SNPs Imputed with 1KG or TOPMed Reference Panels.

Figure S4. Total Sample Pneumonia GWAS QQ Plots.

Table S4. Total Sample β-globin Locus SNP Risk Allele Frequencies by Pneumonia Case/control Status and Imputation Reference Panel.

Table S5. Linkage Disequilibrium Between Selected SNPs in the Total Sample and Cases only.

Figure S5. Genotyping-matched Subset Pneumonia GWAS Manhattan and QQ Plots by Imputation Reference Panel.

Figure S6. Genotyping-matched Subset β-globin Locus Plots of Plots of Pneumonia GWAS Results with and without Conditioning on 1KG rs334 dosages.

Figure S7. Total Sample β-globin Locus Plots of Pneumonia GWAS Results with and without Conditioning on rs2226952 Dosages.

Table S6. Total Pneumonia GWAS Results for Selected β-globin Locus SNPs with and without Conditioning on rs2226952.

Figure S8. Genotyping-matched Subset β-globin Locus Plots of Pneumonia GWAS Results with and without Conditioning on 1KG rs2226952 Dosages.

Table S7. Genotyping-matched Subset Pneumonia GWAS Results for Selected β-globin SNPs with and without Conditioning with rs334 and rs2226952.

Table S8. TOPMed Imputed Total Sample Pneumonia GWAS Genome-wide Significant SNP after Conditioning with 1KG rs334 Dosages.

Figure S9. Total Sample Locus Plot of GWAS Genome-wide Significant SNP after Conditioning on 1KG rs334 Dosages.

Table S9. Pneumonia GWAS Results for Selected β-globin SNPs with and without Individuals with Sickle Cell Disease.

Table S1. Number of Variants used for Imputation and Obtained from Imputation by Pneumonia Case/control Dataset and Imputation Reference Panel

Dataset		Imputation Information			Imputed SNPs, N			Imputed SNPs with MAC≥ 10, N	
Name	Array	SNPs Used, N	Samples, N	Reference Panel	Total	MAC < 10	MAC ≥ 10	MAF ≤ 0.01	MAF > 0.01
Total	168K combined	168,220	2,530	TOPMed	65,908,902	42,047,233	23,861,669	8,566,724	15,294,945
Total	168K combined	168,220	2,530	1KG	24,228,518	4,589,186	19,639,332	5,565,104	14,074,228
Genotyping-matched subset	MEGA	834,828	1,000	TOPMed	65,908,902	47,271,085	18,637,817	3,324,520	15,313,297
Genotyping-matched subset	MEGA	834,828	1,000	1KG	24,228,518	7,704,592	16,523,926	2,436,328	14,087,598

Input to, and output of, imputation using 1KG or TOPMed reference panels of total or genotyping-matched datasets. Imputed SNP numbers are after removing monomorphic, non-biallelic SNPs and SNPs with estimated imputation quality $\widehat{r^2}$ < 0.3. SNP, single nucleotide polymorphism; MAC, minor allele count; MAF, minor allele frequency

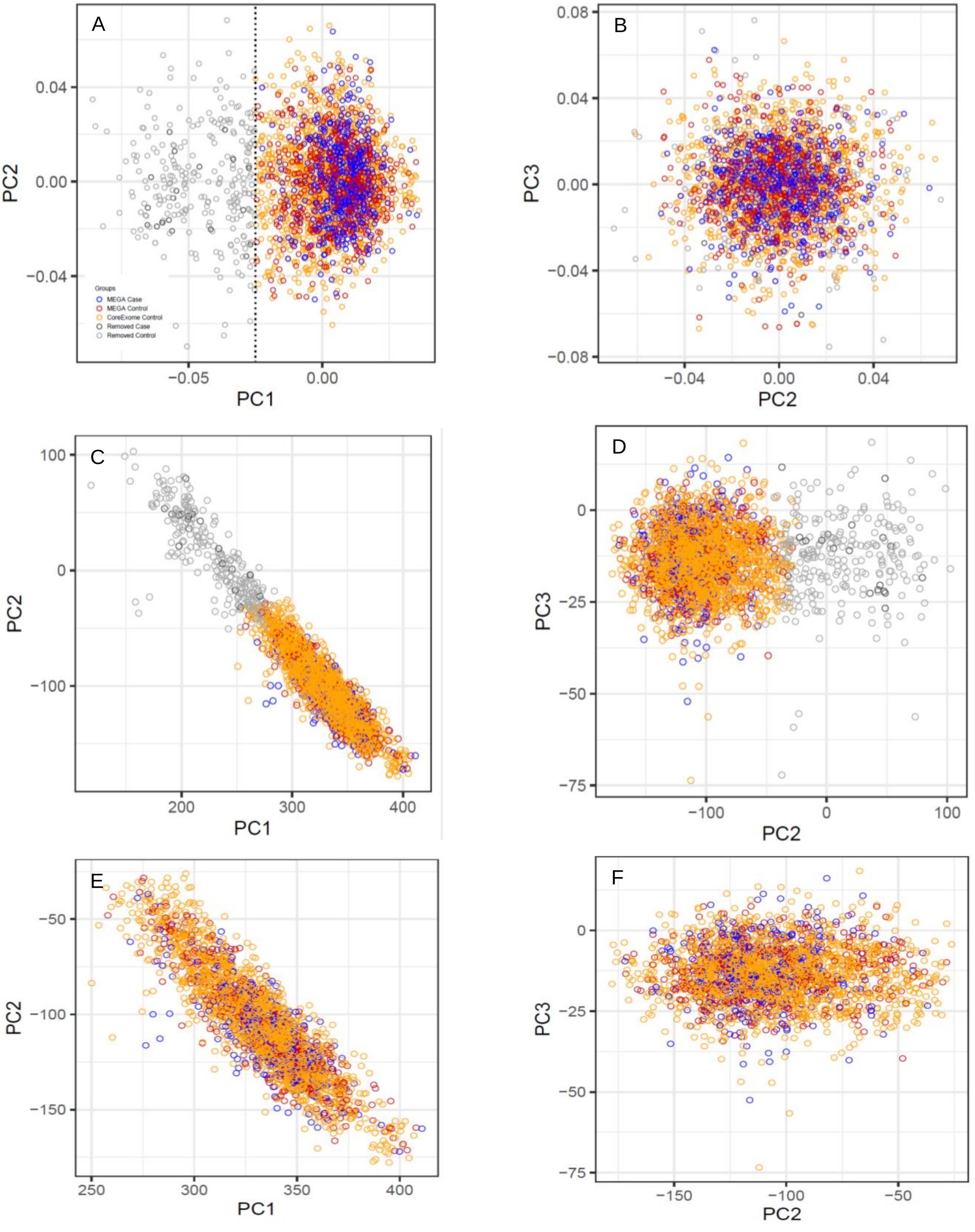

Figure S1. Genotype Principal Components Analysis (PCA) from PLINK (A-B) and Genetic Ancestry PCA from LASER/TRACE (B-D) of Total Sample before (A-D) and after (E-F) removing samples with PC1 < -0.025. Pneumonia samples denoted by case/control status, genotyping array, and if removed from further analysis. A, C and E, PC1 vs PC2; B, D and E, PC2 vs PC3; PC, principal component.

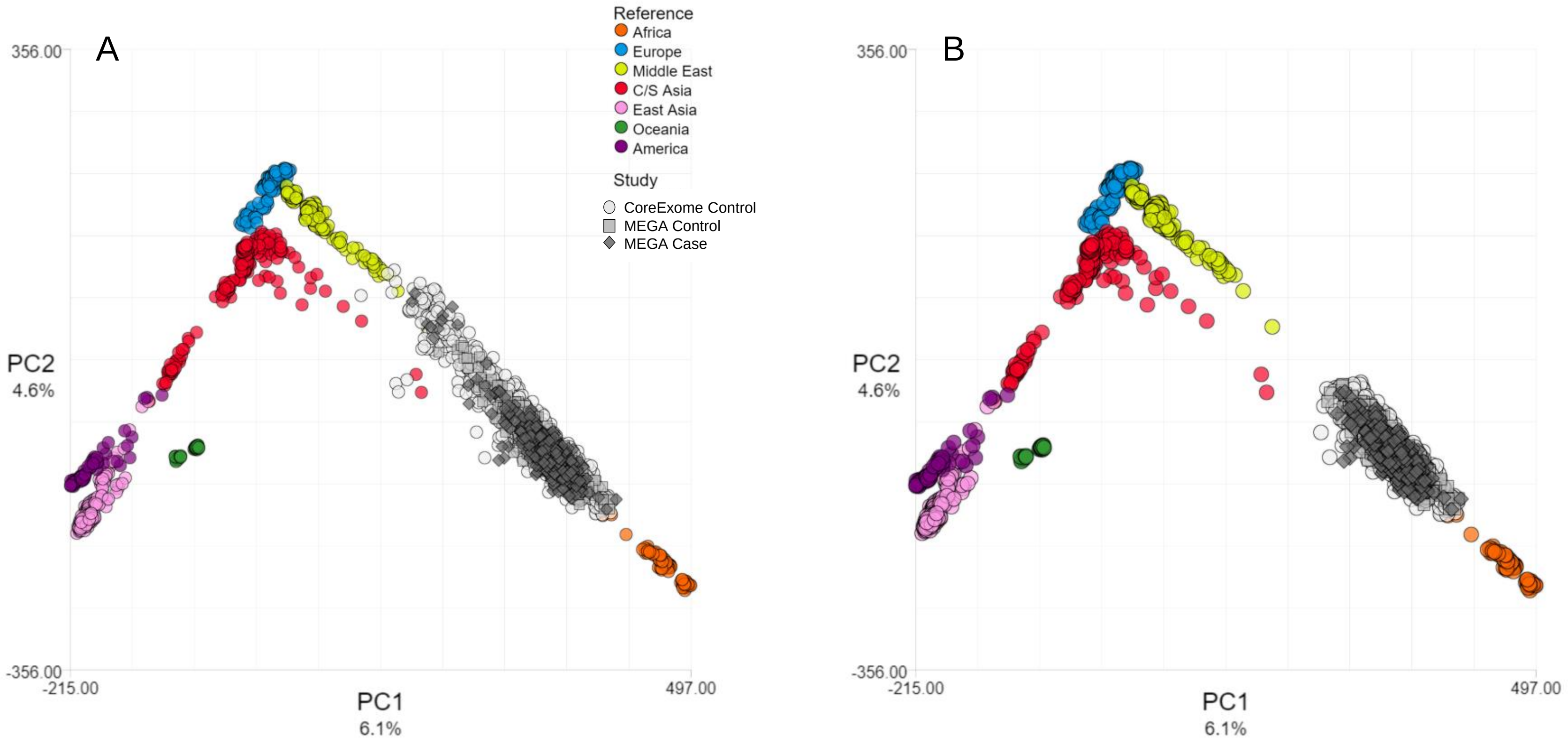

Figure S2. Genetic Ancestry Principal Components from LASER/TRACE Analysis in the Total Sample and Human Genome Diversity Project (HGDP) Samples. Pneumonia study samples denoted by case/control status and genotyping array. A, total sample prior to removing samples for PC1 < -0.025 in PLINK PCA; B, final total sample set. A and B, PC1 vs PC2; PC, principal component.

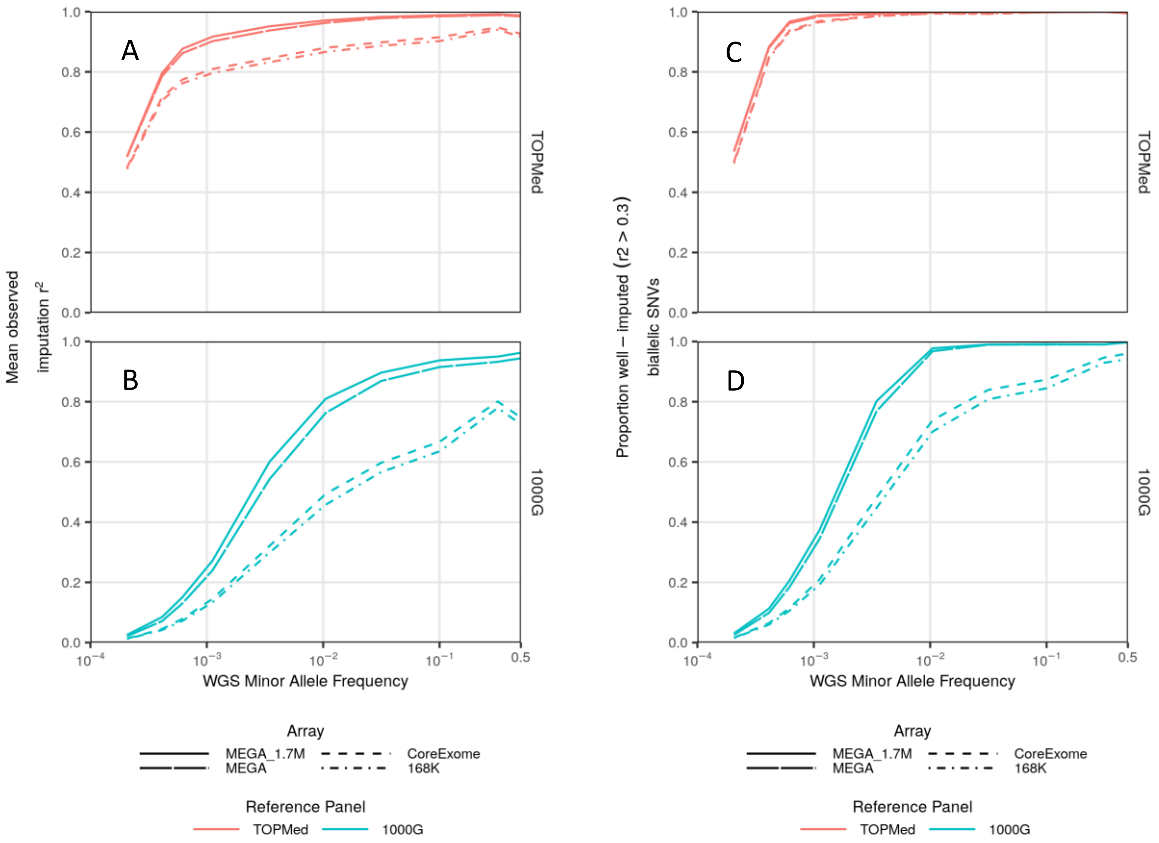

Figure S3. Observed Imputation Quality of InPSYght Genotypes Observed imputation correlation r^2 of whole genome sequencing (WGS) InPSYght genotypes with imputed genotype dosages from TOPMed (A,C) or 1KG (B, D) reference panels using chr11 SNPs. Array genotypes from MEGA (835K SNPs), total dataset (168K SNPs), and CoreExome array (188K SNPs). A & B, mean observed imputation r^2 for a given array vs InPSYght WGS minor allele frequency (MAF); C & D, proportion of imputed biallelic single nucleotide variants (SNVs) with observed $r^2 > 0.3$ by InPCYght WGS MAF.

Table S2. Average Effective Sample Size Adjusted for Imputation

Sample Name	Number of Variants in Imputation	Total Sample Size, N	Effective Sample Size, N_effective	Average Effective Sample Size Adjusted for Imputation, N_effective_imputation			
				MAF >0.01		rs334	
				1 KG	TOPMed	1 KG	TOPMed
Total	168,220	2530	1562	968	1406	1125	NA
Genotyping-matched	834,828	1000	999	879	979	749	NA

Effective samples size adjusted for mean observed chromosome 11 SNP imputation quality is (r^2) x (N_effective_imputation). Effective samples size adjusted for rs334 observed imputation quality is (r^2) x (N_effective_imputation_rs334). MAF, minor allele frequency

Table S3. Estimated and Observed Imputation Quality of β-globin SNPs Imputed with 1KG or TOPMed Reference Panels

SNP	hg38 Position	Estimated Imputation Quality ($\hat{r}^2$) for Indicated Samples, Genotyping Array Dataset and Reference Panel				Observed Imputation Quality (r^2) for Indicated Samples, Genotyping Array Dataset and Reference Panel			
		Total (168K SNPs)		Genotyping-matched subset (835K SNPs)		Total (168K SNPs)		Genotyping-matched subset (835K SNPs)	
		1KG	TOPMed	1KG	TOPMed	1KG	TOPMed	1KG	TOPMed
rs334	5227002	0.81	NA	0.92	NA	0.72	NA	0.75	NA
rs33930165	5227003	0.66	0.92	0.98	1.00	0.55	0.87	0.92	0.98
rs2226952	5285924	0.75	0.94	0.97	1.00	0.59	0.90	0.97	0.99

NA, not applicable as rs334 is not imputed in TOPMed; SNP, single nucleotide polymorphism

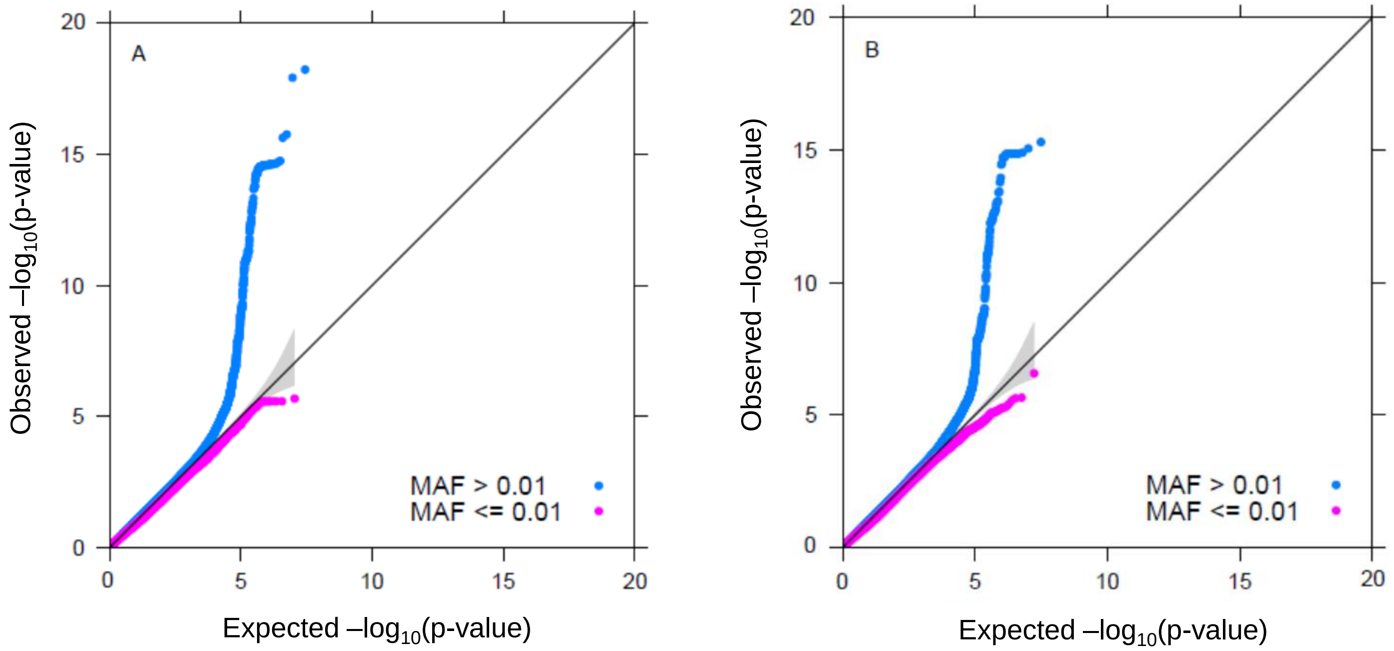

Figure S4. Total Sample Pneumonia GWAS QQ Plots QQ Plots of total sample pneumonia GWAS of single nucleotide polymorphisms (SNPs) imputed with 1KG (A) and TOPMed (B). Firth logistic regressions for SNPs with allele count ≥ 10. Plotted by minor allele frequency (MAF) bin. Solid line y=x.

Table S4. Total Sample β-globin Locus SNP Risk Allele Frequencies by Pneumonia Case/control Status and by Imputation Reference Panel

SNP	Risk/non-risk alleles	Position	Risk Allele Frequencies							
			1KG Reference Panel				TOPMed Reference Panel			
			Cases: MEGA	Controls: MEGA	Controls: CoreExome	Controls: MEGA & CoreExome	Cases: MEGA	Controls: MEGA	Controls: CoreExome	Controls: MEGA & CoreExome
rs334	A/T	5,227,002	0.16	0.059	0.063	0.062	NA	NA	NA	NA
rs33930165	T/C	5,227,003	0.033	0.013	0.012	0.012	0.035	0.0086	0.011	0.010
rs2226952	G/T	5,285,924	0.23	0.12	0.13	0.13	0.24	0.12	0.14	0.13

All samples were imputed with 168K array. The *HBB* gene is transcribed from the reverse strand of the genome (alleles called on the forward strand). Position, genome build GRCh38/hg38 base position on chromosome 11; SNP, single nucleotide polymorphism; NA, not applicable as rs334 is not imputed in TOPMed.

Table S5. Linkage Disequilibrium Between Selected SNPS in the Total Sample and Cases only

	Linkage disequilibrium in total sample/cases only (r^2 unshaded, D' shaded)					
	1KG			TOPMed		
	rs334	rs33930165	rs2226952	rs334*	rs33930165	rs2226952
rs334		0.01/0.03	0.27/0.33		0.01/0.03	0.19/0.25
rs33930165	0.24/0.42		0.06/0.11	0.17/0.42		0.06/0.07
rs2226952	0.64/0.62	0.92/1.0		0.64/0.63	0.83/0.82	

Linkage disequilibrium r^2 (unshaded) and D' (shaded) between rs334, rs33930165, and rs2226952 by imputation reference panel in the total sample/cases only. *LD to rs334 in TOPMed based on 1KG rs334 dosages

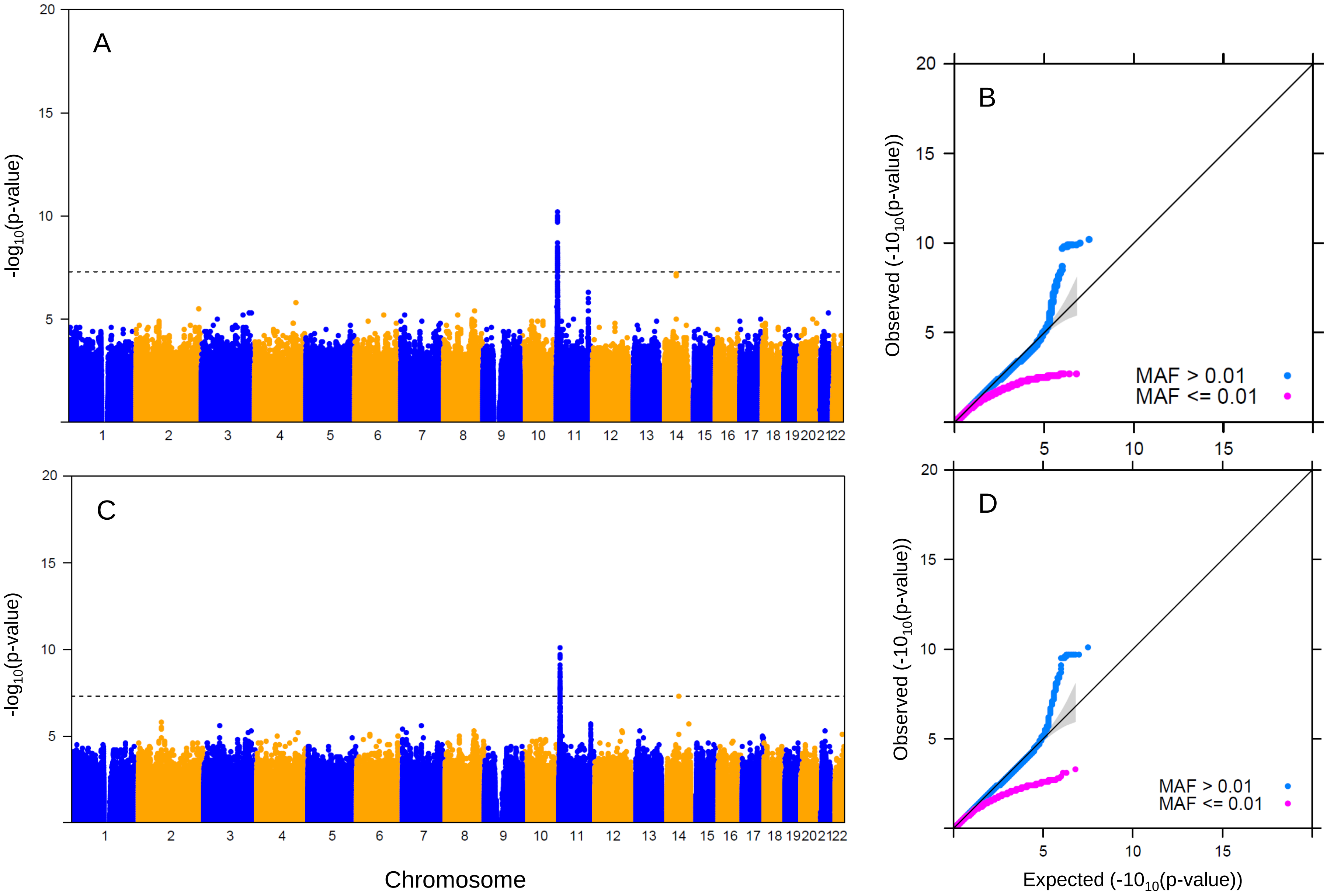

Figure S5. Genotyping-matched Subset Pneumonia GWAS Manhattan and QQ Plots by Imputation Reference Panel
Manhattan (A, C) and QQ plots (B, D) of genotyping-matched subset imputed with TOPMed (top) and 1KG (bottom). Firth logistic regressions of SNPs with allele counts ≥ 10. Dotted line, $p = 5 \times 10^{-08}$; solid line $y = x$.

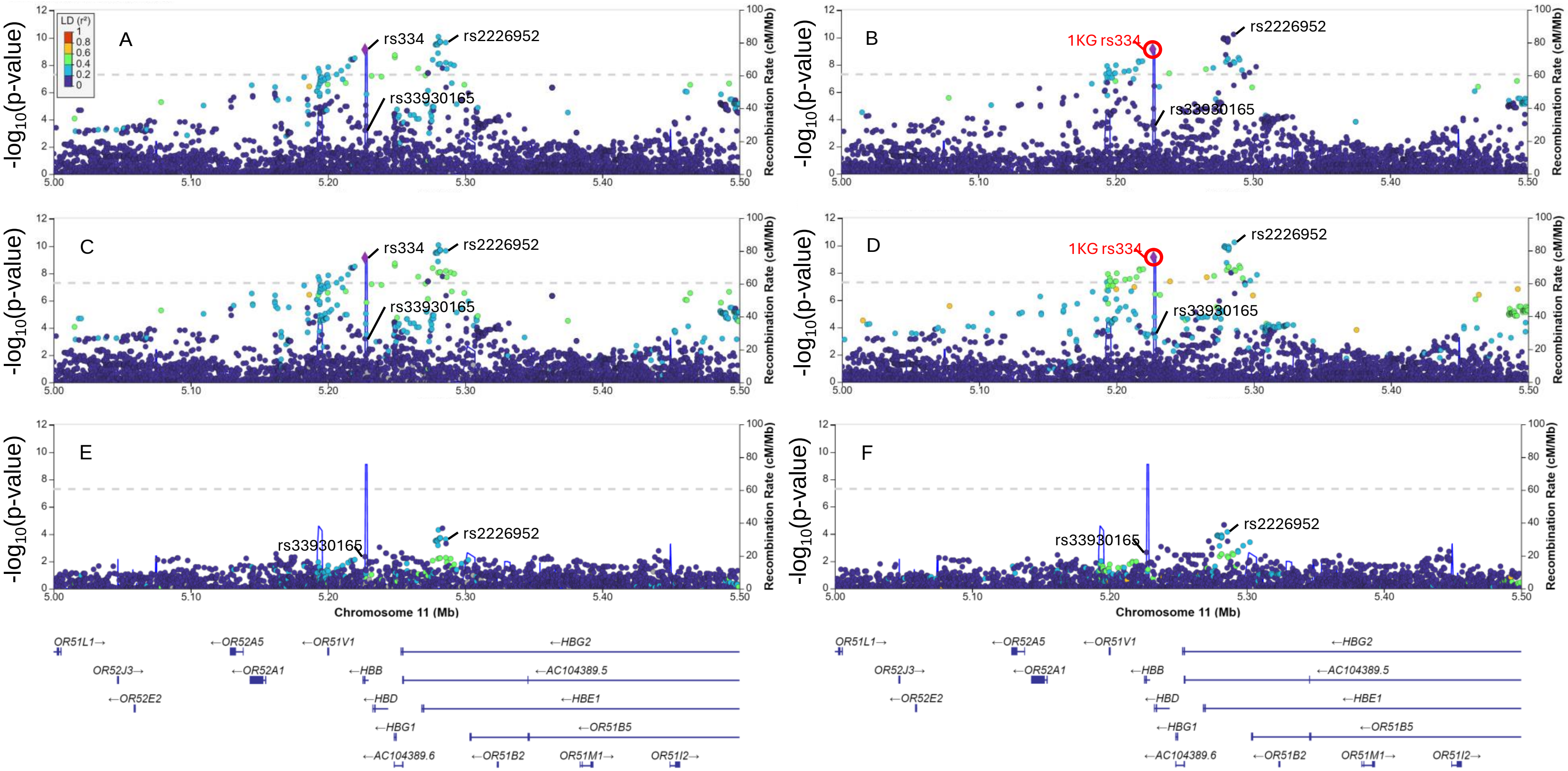

Figure S6. Genotyping-matched Subset β-globin Locus Plots of Pneumonia GWAS Results with and without Conditioning on 1KG rs334 dosages (colored by r^2 to 1KG imputed rs334) Plots of β-globin locus for the genotyping-matched subset with 1KG (left) and TOPMed (right) imputation. r^2 to rs334 (from 1KG imputation) in all samples (A, B), and in cases only (C-F). GWAS analysis conditioned on rs334 1KG dosages shown in E-F. Variant and gene positions shown in build hg38; 1KG rs334 added to TOPMed plots for comparison purposes; dotted line, 5×10^{-08}.

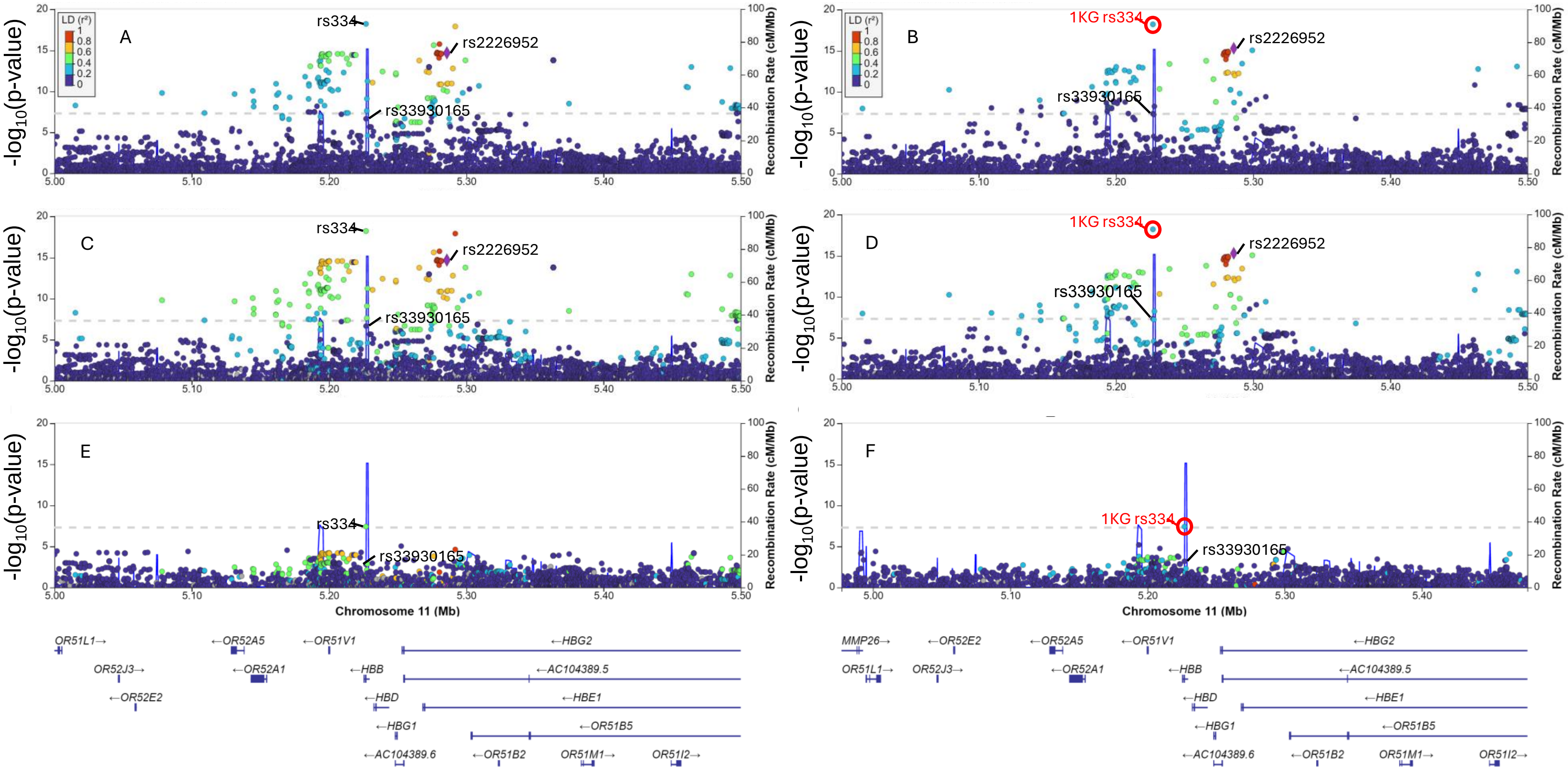

Figure S7. Total Sample β-globin Locus Plots of Pneumonia GWAS Results with and without Conditioning on rs2226952 Dosages (colored by r^2 to 1KG imputed rs2226952) Plots of β-globin locus for the genotyping-matched subset with 1KG (left) and TOPMed (right) imputation. r^2 to rs2226952 in all samples (A, B), and in cases only (C-F). GWAS analysis conditioned on rs2226952 dosages shown in E-F. Variant and gene positions shown in build hg38; 1KG rs334 added to TOPMed plots for comparison purposes; dotted line, 5 x 10^{-08}.

Table S6. Total Sample Pneumonia GWAS Results for Selected β-globin Locus SNPs with and without Conditioning on rs2226952

SNP	Risk/Non-risk Alleles	1KG				TOPMed			
		Unconditioned		Conditioned on rs2226952		Unconditioned		Conditioned on rs2226952	
		OR (95% CI)	p-value	OR (95% CI)	p-value	OR (95% CI)	p-value	OR (95% CI)	p-value
rs334	A/T	2.76 (2.21-3.46)	5.9×10^{-19}	2.10 (1.62-2.72)	3.8×10^{-08}	NA	NA	2.09[a] (1.61-2.70)	1.9×10^{-08}
rs33930165	T/C	4.44 (2.52-7.82)	2.2×10^{-07}	2.59 (1.45-4.65)	1.5×10^{-03}	3.89 (2.38-6.35)	4.7×10^{-08}	2.42 (1.46-4.01)	6.5×10^{-04}
rs2226952	G/T	2.29 (1.86-2.81)	2.0×10^{-15}	--	--	2.14 (1.78-2.57)	5.1×10^{-16}	--	--

Total dataset imputed with 1KG or TOPMed reference panels and analyzed using Firth logistic regression with and without conditioning on rs2226952 dosage. The *HBB* gene is transcribed from the reverse strand of the genome (alleles called on the forward strand). [a]1 KG rs334 OR, 95% CI, and p-value after conditioning with TOPMed rs2226952 gene dosages; SNP, single nucleotide polymorphism; OR, odds ratio; CI, confidence interval; NA, not applicable as rs334 is not imputed in TOPMed

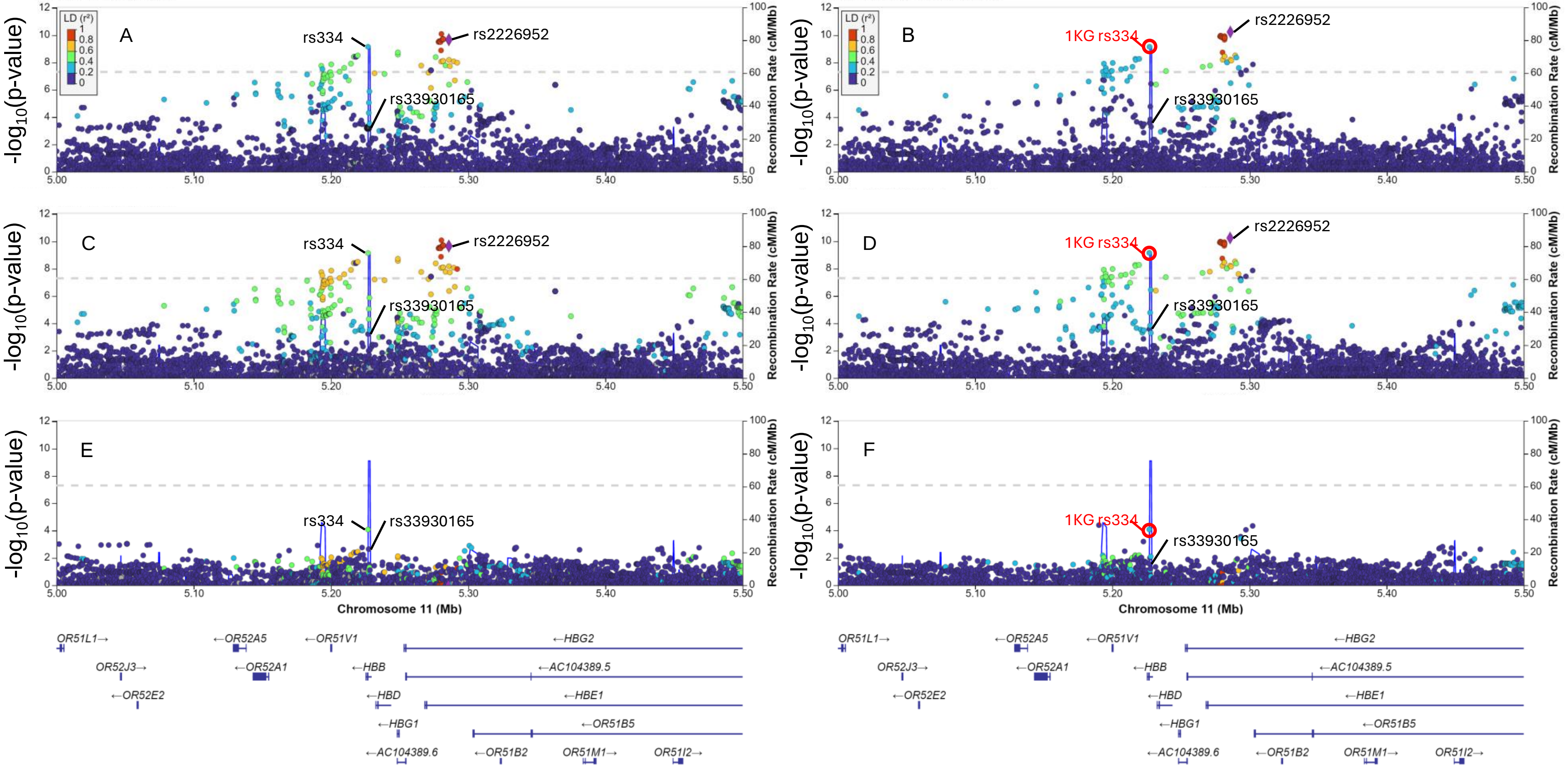

Figure S8. Genotyping-matched Subset β-globin Locus Plots of Pneumonia GWAS Results with and without Conditioning on 1KG rs2226952 Dosages (colored by r^2 to 1KG imputed rs2226952) Plots of β-globin locus for the genotyping-matched subset with 1KG (left) and TOPMed (right) imputation. r^2 to rs2226952 in all samples (A-B), and in cases only (C-F). GWAS analysis conditioned on rs2226952 dosages shown in E-F. Variant and gene positions shown in build hg38; 1KG rs334 added to TOPMed plots for comparison purposes; dotted line, 5×10^{-08}.

Table S7. Genotyping-matched Subset Pneumonia GWAS Results for Selected β-globin SNPs with and without Conditioning with rs334 or rs2226952

Imputation Reference Panel	SNP	Risk/Non-risk alleles	Unconditioned		Conditioned on rs334		Conditioned on rs2226952	
			OR (95% CI)	p-value	OR (95% CI)	p-value	OR (95% CI)	p-value
1 KG	rs334	A/T	2.72 (1.98-3.75)	7.1×10^{-10}	--	--	2.03 (1.42-2.90)	8.4×10^{-05}
	rs33930165	T/C	3.26 (1.65-6.42)	6.1×10^{-04}	2.72 (1.36-5.44)	4.5×10^{-03}	1.98 (0.98-4.02)	5.7×10^{-02}
	rs2226952	G/T	2.18 (1.71-2.78)	2.2×10^{-10}	1.67 (1.27-2.20)	2.4×10^{-04}	--	--
TOPMed	rs334	A/T	NA	NA	NA	NA	NA	NA
	rs33930165	T/C	3.73 (1.83-7.61)	2.7×10^{-04}	3.08 (1.49-6.36)	2.24×10^{-03}	2.29 (1.09-4.79)	2.7×10^{-02}
	rs2226952	G/T	2.22 (1.75-2.82)	5.8×10^{-11}	1.72 (1.32-2.26)	7.15×10^{-05}	--	--

Genotype-match subset imputed using 1KG or TOPMED reference panels and analyzed using Firth logistic regression without and with conditioning on 1KG-imputed rs334 or rs2226952 (from respective imputations). The *HBB* gene is transcribed from the reverse strand of the genome (alleles called on the forward strand). SNP, single nucleotide polymorphism, OR, odds ratio; CI, confidence interval; NA, not applicable as rs334 in not imputed with TOPMed.

Table S8. TOPMed imputed Total Sample Pneumonia GWAS Genome-wide Significant SNP after Conditioning with 1KG rs334 Dosages

SNP	Risk/Non-risk alleles	1KG					TOPMed				
		Unconditioned		Conditioned on rs334		$\widehat{r^2}$	Unconditioned		Conditioned on rs334		$\widehat{r^2}$
		OR (95% CI)	p-value	OR (95% CI)	p-value		OR (95% CI)	p-value	OR (95% CI)	p-value	
rs12137603	A/C	1.49 (1.06 - 2.09)	0.020	1.48 (1.05 - 2.09)	0.023	0.42	1.81 (1.45 - 2.25)	1.6×10^{-07}	1.88 (1.50 - 2.35)	4.2×10^{-08}	0.86

Total sample imputed with 1 KG TOPMed reference panel Firth logistic regression result upon conditioning with 1KG rs334 dosages. rs12137603 in genome build GRCh38/hg38 at position 240,484,996 in chromosome 1. SNP, single nucleotide polymorphism; $\hat{r}^2$, estimated imputation quality for given imputation; OR, odds ratio; CI, confidence interval.

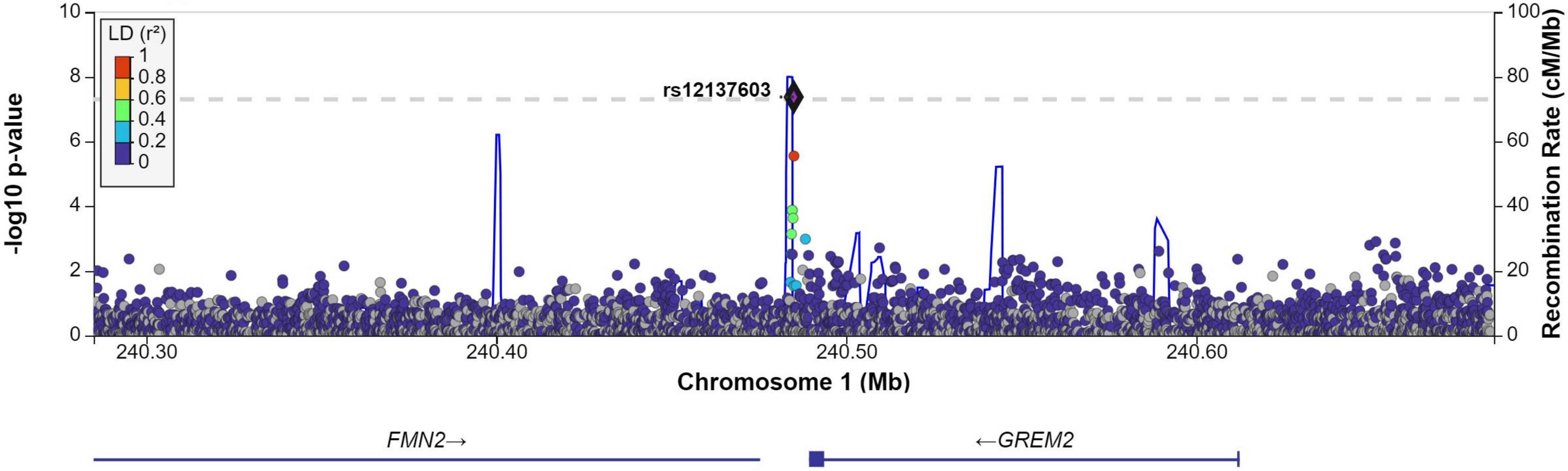

Figure 9S. Total Sample Locus Plot of GWAS Genome-wide Significant SNP after Conditioning on 1KG rs334 Dosages. TOPMed imputation with LD to rs12137603 from African population in 1KG reference panel. Variant and gene positions shown in build hg38, dotted line, 5×10^{-08}.

Table S9. Pneumonia GWAS Results for Selected β-globin SNPs with and without Individuals with Sickle Cell Disease

Imputation Reference Panel	SNP	Risk/Non-risk alleles	Total Sample		Without Individuals with SCD	
			OR (95% CI)	p-value	OR (95% CI)	p-value
1 KG	rs334	A/T	2.76 (2.21-3.46)	5.9×10^{-19}	1.24 (0.88-1.76)	0.22
	rs33930165	T/C	4.44 (2.52-7.82)	2.2×10^{-07}	3.08 (1.61-5.90)	6.2×10^{-04}
	rs2226952	G/T	2.29 (1.86-2.81)	2.0×10^{-15}	1.49 (1.17-1.90)	1.1×10^{-03}
TOPMed	rs334	A/T	NA	NA	NA	NA
	rs33930165	T/C	3.89 (2.38-6.35)	4.7×10^{-08}	2.78 (1.58-4.85)	3.2×10^{-04}
	rs2226952	G/T	2.14 (1.78-2.57)	5.1×10^{-16}	1.54 (1.25-1.91)	5.0×10^{-05}

Total sample imputed using 1KG or TOPMED reference panels. Total sample with and without individuals with SCD analyzed using Firth logistic regression. The *HBB* gene is transcribed from the reverse strand of the genome (alleles called on the forward strand). SNP, single nucleotide polymorphism, SCD, sickle cell disease; OR, odds ratio; CI, confidence interval; NA, not applicable as rs334 in not imputed with TOPMed
