# Supplementary material for: Variants in the β-globin locus are associated with pneumonia in African American children
Source: HGG Adv. 2024 Oct 22;6(1):100374. doi: 10.1016/j.xhgg.2024.100374 (PMC11664401; doi:10.1016/j.xhgg.2024.100374)
Supplement: Document S2. Article plus supplemental information [file mmc2.pdf]

HGG Advances

ARTICLE

Variants in the β-globin locus are associated with pneumonia in African American children

Nadine L.N. Halligan,[1] Sarah C. Hanks,[2] Karen Matsuo,[2] Taylor Martins,[2,4] Sebastian Zöllner,[2,3] Michael W. Quasney,[1] Laura J. Scott,[2,5,*] and Mary K. Dahmer[1,*]

Summary

In African American adults, the strongest genetic predictor of pneumonia appears to be the A allele of rs334, a variant in the β-globin gene, which in homozygous form causes sickle cell disease (SCD). No comparable studies have been done in African American children. We performed genome-wide association analyses of 482 African American children with documented pneumonia and 2,048 African American control individuals using genotypes imputed from two reference panels: 1000 Genomes (1KG) (which contains rs334) and TOPMed (does not contain rs334). Using 1KG imputed genotypes, the most significant variant was rs334 (A allele; odds ratio [OR] = 2.76; 95% CI, 2.21–3.74; $p = 5.9 \times 10^{-19}$); using TOPMed imputed genotypes the most significant variant was rs2226952, found in the β-globin locus control region (G allele; OR = 2.14; 95% CI, 1.78–2.57; $p = 5.1 \times 10^{-16}$). After conditioning on rs334, the most strongly associated variant in the β-globin locus, rs33930165 (T allele, 1KG: OR = 4.09; 95% CI, 2.29–7.29; $p = 1.7 \times 10^{-6}$; TOPMed: OR = 3.58; 95% CI, 2.18–5.90; $p = 4.7 \times 10^{-7}$), which as a compound heterozygote with rs334 A allele, can cause SCD. To compare the power of different sample sets we developed a way to estimate the power of sample sets with different sample sizes, genotype arrays, and imputation platforms. Our results suggest that, in African American children, the strongest genetic determinants of pneumonia are those that increase the risk of SCD.

Introduction

Globally, pneumonia is one of the leading causes of death in children under 5.[1,2] In the US, it is one of the most frequent reasons for hospitalizations in children[3] and one of the most common causes of pediatric deaths.[4] In 2015 a prospective, population-based study reported an incidence of hospitalization for pneumonia in the US of 15.7 per 10,000 children overall and 62.2 per 10,000 in children under 2.[5] A recent NHLBI workshop report highlighted the need to identify genetic variants that influence individuals' susceptibility to, and response to, pneumonia.[6]

An individual's genetic make-up influences their risk of infection. In an early study of adoptees, individuals with a biological parent who had died of infection were 5.8-fold more likely to die of infection.[7] More recently, genome-wide association studies (GWASs) have identified variants associated with infectious disease risk,[8–15] including COVID-19[10,11] and pneumonia.[9,12–15] All existing GWASs of pneumonia have been performed in adults, although one study analyzed self-reported childhood pneumonia.[14] European ancestry-based pneumonia GWASs have identified a small number of loci associated with self-reported or adult medical record-based pneumonia.[9,12,13,15] A BioVu African ancestry-based pneumonia GWAS ($n = 1{,}710$ affected individuals and 13,871 control individuals) identified a coding variant (rs334 A allele) in the β-globin gene (*HBB*) associated with pneumonia[13]; the rs334 association was not detected in the European ancestry-based GWAS, however, the frequency is much lower in those of European ancestry compared with those of African ancestry (ALFA frequency = 0.00006 and 0.003, respectively). *HBB* is the causative gene for sickle cell disease (SCD [MIM: 603903]) and homozygosity for the rs334 A allele is the most common cause of SCD.[16] Epidemiologic studies indicate that individuals with SCD[17] have an increased risk of pneumonia. In contrast, no genome-wide associated variants were identified in the COPDGene African ancestry-based GWAS (157 childhood pneumonia affected individuals and 3,124 control individuals; 882 lifetime pneumonia affected individuals and 2,237 control individuals) in which all participants were smokers.[14]

No GWASs for pneumonia have been performed in children, who might have different genetic risk factors than adults. In addition, African American individuals are under-represented in GWASs.[18] Thus, we aimed to identify genetic risk factors for pneumonia in 482 African American children with documented pneumonia. Because we did not collect control individuals with the affected individuals, we used as control individuals, 2,048 ancestry-matched adult African American individuals from the Michigan Genomics Initiative (MGI) Biobank. Starting

[1]Division of Critical Care Medicine, Department of Pediatrics, University of Michigan, Ann Arbor, MI 48109, USA; [2]Department of Biostatistics and Center for Statistical Genetics, School of Public Health, University of Michigan, Ann Arbor, MI 48109, USA; [3]Department of Psychiatry, University of Michigan, Ann Arbor, MI 48109, USA
[4]Present address: Informatics Division, Business Intelligence Office, Arizona Department of Health Services, Phoenix, AZ, 85007, USA
[5]Lead contact
*Correspondence: ljst@umich.edu (L.J.S.), mkdahmer@med.umich.edu (M.K.D.)
https://doi.org/10.1016/j.xhgg.2024.100374.
© 2024 The Author(s). Published by Elsevier Inc. on behalf of American Society of Human Genetics.
This is an open access article under the CC BY license (http://creativecommons.org/licenses/by/4.0/).

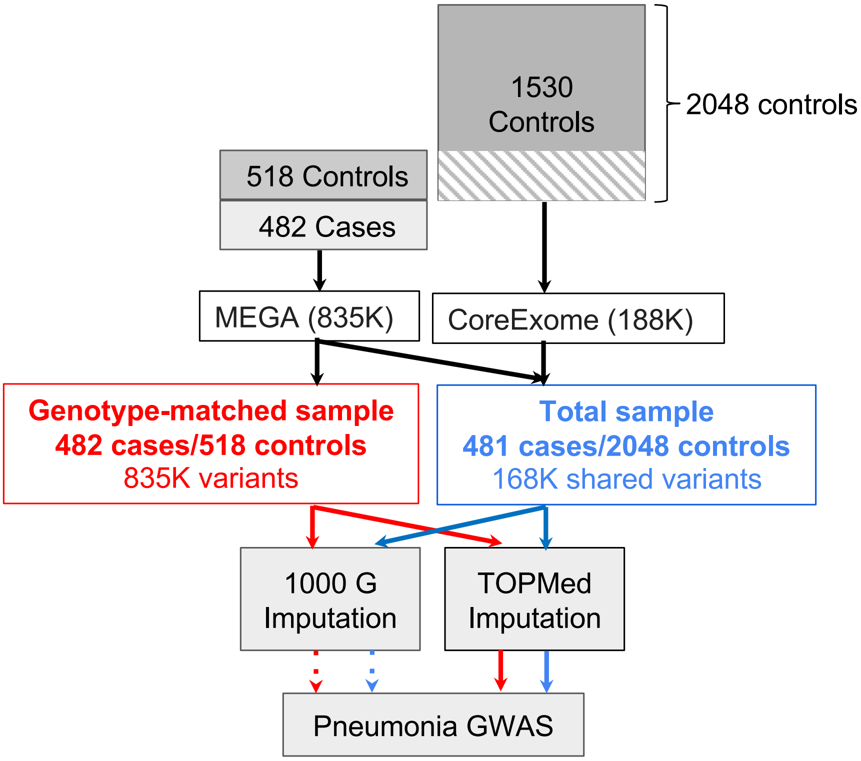

Figure 1. Pneumonia GWAS study flowchart
A total of 2,048 MGI control individuals was genotyped using the CoreExome array. Four hundred and eighty-two affected individuals (referred to as cases in the Figure) and 518 of the MGI control individuals were genotyped using the MEGA array; these samples are referred to as genotype-matched samples (red). The combination of the genotype-matched samples and remaining 1,530 MGI CoreExome genotyped samples (non-hash-marked samples) is referred to as the total sample (blue). Starting with 835K variants for genotype-matched samples and 168K shared variants for the total sample, the samples were imputed using the 1KG (dashed line) and TOPMed (solid line) reference panels. GWASs were performed for the four combinations of samples and imputation panels.

with the genotype data, we imputed variants using the 1000 Genomes (1KG) reference panel (which includes rs334)[19] (IGSR: 1000 Genomes phase 3 release) and the TOPMed panel (which does not include rs334)[20] (TOPMed: version TOPMed-r2) and tested for genetic variant-pneumonia association. To aid in assessment of our findings, we evaluated the quality of variants imputed from the 1KG and TOPMed reference panels using African American whole-genome sequence data from a non-pneumonia study and estimated the power to detect associations in sample sets of different sizes, genotype arrays, and imputation panels.

Subjects and methods

Figure 1 provides a flowchart of the study including the study design, samples, genotyping, imputation, and analysis.

Design and study participants

The report follows the guidelines for genetic association studies outlined by the Strengthening the Reporting of Genetic Association Studies reporting guidelines.[21] The Institutional Review Boards from each institution approved the study and the procedures followed were in accordance with the ethical standards of the responsible committee on human experimentation.

Affected individuals

We performed a affected individual-control individual GWAS using as affected individuals a subset of children who were prospectively enrolled into a study of community-acquired pneumonia at four tertiary care pediatric hospitals (LeBonheur Children's Hospital, Memphis, TN; Children's Memorial Hospital, Chicago, IL; Children's Hospital of Wisconsin, Milwaukee, WI; CS Mott Children's Hospital, Ann Arbor, MI) between 2004 and 2017. Written informed consent was obtained from parents or guardians of those enrolled in 2008 or later. Samples between 2004 and 2007 were from discarded blood and consent was either obtained in person or over the phone. In this analysis, we included as pneumonia affected individuals who: (1) self (or parent)-identified as African American; (2) were aged 10 days to 18 years; (3) were seen in the emergency department, or admitted to the general pediatric ward or intensive care unit; (4) were determined to have community-acquired pneumonia; and (5) had a complete blood count and chest radiograph as part of their care. Community-acquired pneumonia was defined as described previously[22,23]: onset of illness (symptoms $<$ 14 days), presence of a new infiltrate on chest radiograph (confirmed by a radiologist), and clinical features suggestive of pneumonia. These clinical features included presence of at least two of the following: tachypnea (respiratory rate >2 SD from the mean for age), dyspnea, hypoxemia (pulse oximetry <94% on room air on initial evaluation and without known mixing heart lesion), cough, or abnormal finding on auscultation of the lungs. In addition, patients were required to have at least one of the following: (1) temperature $\leq$ 36°C (individuals in shock) or $\geq$38.5°C, (2) peripheral white blood cell count $\leq$4,500/mL or $\geq$10,000/mL, or (3) $\geq$15% immature neutrophils.

Control individuals

Control individuals were chosen from the MGI, a repository of DNA data, biospecimens, and electronic health records from patients $\geq$18 years of age undergoing elective surgery or a diagnostic procedure at the University of Michigan Hospital (Fritsche et al.[24]). Written informed consent was obtained from patients contributing to the MGI repository. The initial inclusion criteria were: (1) self-identified as African American and (2) genotyped on a custom Illumina Infinium Core-Exome24 microarray, "UM_HUNT_Biobank_11788091_A1" (Illumina, San Diego, CA), subsequently referred to as the CoreExome microarray. We had no information on childhood pneumonia in control individuals.

SCD classification

For affected individuals, we defined them as having SCD if they were identified as having SCD in the medical chart (n = 59). For

control individuals, we used electronic health records to identify those with SCD. If an individual had no codes for sickle cell trait (being a carrier of an SCD variant, but not having SCD [coded as 282.5]), we defined SCD as having one or more SCD ICD-9-CM codes (282.41, 282.42, or 282.6x). If an individual had ICD-9 codes for both SCD and sickle cell trait, we defined SCD as having a value of >90% for (number of encounters with an SCD code)/(number of encounters with SCD code + number of additional encounters with sickle cell trait code).

Comorbid conditions

Comorbid conditions in pneumonia affected individuals were determined from review of the medical chart with neurological disorders defined as those with seizures or developmental delay. Comorbid conditions in control individuals were defined using the following ICD-9 codes. For asthma, all subcategories under 493 were used. For neurological disorders, all subcategories under 330, 331, 332, 334, 335, 340, 341, 342, 343, 344, 345, and 347 were used together with the subcategories 333.4, 333.5, 333.92, 336.2, 348.1, 348.30, 348.31, 348.39, 438.2–438.5, 780.3, and 784.3. Chronic lung disease was defined by all subcategories under 490, 491, 492, 494, 495, 496, 500–505, and subcategories 506.4, 508.1, 518.83, and 748.61.

Array genotyping

All control individuals were genotyped previously on the Illumina Infinium CoreExome-24 bead array as described.[24] Pneumonia affected individuals ($n = 531$) and 549 of the control individuals were concurrently genotyped at the University of Michigan Biomedical Research Advanced Genomics Core on the Illumina Multi-Ethnic Genotyping (MEGA)_Consortium_v2 BeadChip Array (referred to as the MEGA array), which was an early version of the current commercially available MEGA array. We chose the MEGA array because it was specifically designed to capture genotype variability in multiple ancestries.[25] We refer to the affected individuals and control individuals genotyped on the MEGA array as the genotyping-matched sample. We refer to the sample containing the affected individuals, control individuals genotyped on the MEGA array, and control individuals genotyped only on the CoreExome array as the total sample.

For affected individuals, DNA was extracted from blood samples using the Wizard Genomic DNA Purification Kit (catalog no. A1120, Promega, Madison, WI) and stored at −80°C. DNA concentration was determined using the Qubit dsDNA BR Assay (catalog no. Q32853) using the Qubit fluorometer (ThermoFisher Scientific, Waltham, MA). DNA samples for affected individuals and 594 control individuals were randomized and plated in alternate wells in 96-well plates. Two HAPMAP DNA trios (NA12892, NA12878, NA12891, NA19238, NA19240, and NA19239) (Coriell Institute for Medical Research, Camden, NJ) were used to assess genotype discrepancies and Mendelian inconsistency rates. A minimum of one parent/child pair were randomly included in each genotyping plate. The average error rate between duplicates was 0.00001284 and average error rate for the triads was 0.0001650.

Genotyping quality control

Quality control of CoreExome array-genotyped control individuals was performed as reported previously.[24] In brief, genotype calling was performed with Illumina GenomeStudio (module 1.9.4, algorithm GenTrain 2.0). Samples with a call rate <98.5% (in all MGI samples genotyped on the array), an inferred sex that did not match reported gender, or with gonosomal constellations other than XX and XY were excluded. Variants were excluded if they had a call rate <99% or they showed deviation from Hardy-Weinberg equilibrium ($p < 0.0001$) in European ancestry samples which are present in the MGI repository but not used in this analysis.

For the MEGA array genotyped affected individuals and control individuals (genotyping-matched subset), we performed quality control using the same procedures and thresholds as for the CoreExome array, with the exception that we used the Illumina GenomeStudio Genotyping Module (v2.0.3, GenTrain 2.0) with the Population Architecture using Genomics and Epidemiology (PAGE) Consortium PAGE_CIDR_MEGA_cluster_definitions.egt cluster files. Variants were excluded if they had a call rate <99% or they showed deviation from Hardy-Weinberg equilibrium ($p < 10^{-6}$).[26] There were five affected individuals and one control individual who were excluded because they did meet the genotyping call rate criteria. There were three affected individuals removed for miscoded sex. One affected individual was removed for being XO. No variants had differentially missing genotypes between affected individuals and control individuals ($p < 10^{-5}$).

TOPMed and 1KG imputation

Genotype array data for total sample of affected individuals and control individuals genotyped on the CoreExome and the MEGA arrays

To jointly impute and analyze affected individuals (MEGA array) and control individuals (MEGA or CoreExome arrays), we created a list of variants present on both arrays as described by Johnson et al.[27] In brief, SNPs genotyped on the MEGA and CoreExome arrays were adjusted for strand differences (https://www.chg.ox.ac.uk/~wrayner/tools/). We then compared the genotype calls for 218,689 variants common to the 549 control individuals genotyped on both the MEGA and CoreExome arrays; we excluded 168 variants that differed by >1 genotype call. The concordance rate for the remaining variants was 0.99994. We also excluded variants with a <98% call rate or with a minor allele frequency (MAF) < 0.01 determined separately in the MEGA and CoreExome arrays; 168,220 SNPs remained (termed the 168K array).

Imputation and Imputation Server

We performed TOPMed and 1KG reference panel-based imputation of the total sample using genotypes from the 168K array (168,220 variants), and imputation of the genotyping-matched subset using genotypes from the MEGA array (834,828 variants). The TOPMed-r2 reference panel ($n = 97,256$) was used because it has higher average African American imputation quality than the 1KG panel.[20] However, the TOPMed-r2 reference panel does not contain rs334 or variants in high LD with rs334 due to QC exclusions. The 1KG reference panel ($n = 2504$) was used because it contains rs334,[19] a causal variant for SCD, which has been previously reported to be associated with pneumonia.[13] To impute using the TOPMed reference panel we used the TOPMed Imputation Server (TOPMed Version r2 2020 Eagle2.4 phasing, GRCh38/hg38)[20,28–30]; to impute using the 1KG reference panel, we used the Michigan Imputation server (1KG phase 3, version 5 reference panel, Eagle2.4 phasing, GRCh37/hg19).[28] 1KG build GRCh37 SNPs were lifted over to build GRCh38 for display purposes. Following imputation, we retained bi-allelic SNPs with estimated $\widehat{r^2} > 0.3$ in the given imputed dataset (Table S1).

Evaluation of imputation quality using whole-genome sequencing data

We independently evaluated the quality of variant imputation for each of the genotype arrays and reference panel combinations

used in this study in a separate set of 2,429 whole-genome sequenced (WGS) African American individuals from the InPSYght study as described in Hanks et al.[31] In brief, we used chromosome 11 WGS data as our gold standard genotype data. We created subsets of the InPSYght WGS variants corresponding to the chromosome 11 variants present in the 168K array, the MEGA array, or the CoreExome array. We phased InPSYght individuals with variants from each array using Eagle2.4.1 and imputed genotypes using Minimac4 on the Michigan Imputation Server[28] with (1) the 1KG Genomes Phase 3 ($n = 2{,}504$) and (2) a modified TOPMed version r2 ($n = 88{,}804$)[31] reference panels. We calculated the observed imputation r^2 for each variant as the squared Pearson correlation coefficient between the imputed genotype dosages and the sequence-based genotypes. We assigned $r^2 = 0$ for any variant that was present in the sequenced individuals but absent from the reference panels and thus was not imputed. For each array and reference panel combination, we calculated the average observed imputation r^2 (mean r^2) for all variants with MAF > 0.01 in the InPSYght sample.

Calculation of effective sample size when using imputed data

We calculated the effective affected individual-control individual sample size for each sample set as $N_{\text{effective}} = \frac{4}{\left(\frac{1}{N_{affected\_individuals}} + \frac{1}{N_{control\_individuals}}\right)}$. We calculated the effective sample size adjusted for imputation quality as $N_{\text{effective\_imputation}} = N_{\text{effective}} \times \text{mean } r^2$. We calculated the effective sample size adjusted for rs334 imputation quality as $N_{\text{effective\_imputation\_rs334}} = N_{\text{effective}} \times r^2_{rs334}$.

Genetic relatedness

We estimated pairwise individual relatedness in the total sample using SNPs in common between the MEGA and CoreExome arrays using KING.[32] We removed individuals so that no pair of individuals had a kinship coefficient >0.088; for related affected individual-control individual pairs we retained the affected individual, otherwise we retained the individual with the higher call rate.

Principal-component-based sample exclusions

We performed principal-component analysis on the total genotype dataset (PLINK v.1.9[26]); we excluded SNPs in regions of high LD[33] and pruned variants to r^2 >0.05 (in PLINK v.1.9 –indep-pairwise 100 10 0.05). We first removed participants >4 standard deviations from the mean for any of the first 10 principal components leaving 2,802 subjects (501 affected individuals and 2,301 control individuals). We removed an additional 272 samples (19 affected individuals, 253 control individuals) with PC1 < −0.025 based on their clustering in a PC1 vs. PC2 plot (Figures S1A and S1B); these were also outliers in a LASER/TRACE ancestry estimation plot (Figures S1C, S1D, and S2A).[34,35] The final dataset had 2,530 individuals (482 affected individuals and 2,048 control individuals) (Figures S1E, S1F, and S2B).

Firth logistic regression

In the total and the genotyping-matched datasets, we tested imputed variants with minor allele count ≥10. We tested for association of documented childhood pneumonia with imputed genotype dosage (TOPMed or 1KG imputation) using Firth logistic regression[36] (PLINK v.2.3a), adjusting for two genotype principal components.[37] To test for independence of association signals at the β-globin gene locus, we performed conditional logistic regression, including one of the following variants as covariates in the model: 1KG-based dosages for rs334 (lead signal in 1KG imputation total sample), or 1KG- or TOPMed-based dosages for rs2226952 (lead signal in the TOPMed imputation). To test for independence of the effects of rs344 and SCD on the risk of pneumonia we included SCD status as a covariate in the Firth logistic regression. To further test for the effect of the heterozygous form of the SCD A allele on pneumonia risk, we removed individuals with SCD (removed individuals homozygous for the rs334 A allele and compound heterozygotes) and performed the Firth logistic regression.

Visualization

We visualized the pneumonia association results and the dataset-specific LD between rs334 or rs2226952 and surrounding variants in our datasets using LocusZoom.[38] We calculated the LD (r^2) between rs334 or rs2226952 and variants within +/− 1 Mb in the total imputed dataset and separately in affected individuals only (PLINK 1.9). rs334 is only present in the 1KG data; thus, we used the 1KG rs334 dosages to assess LD of rs334 with TOPMed imputed variants.

Gene set analysis

We tested for gene set enrichment using MAGMA, using the Firth logistic regression *p* values from the total sample TOPMed-imputed dosages.[39] We used the MAGMA default settings except that we extended gene boundaries by +/− 2 kb. We used imputation-based hard-called genotypes to estimate SNP linkage disequilibrium (PLINK v.1.9). We performed gene set analysis using the SNP-wise model with two-sided *p* values using (1) the canonical pathways gene sets (2,922 gene sets) and (2) Gene Ontology (GO) gene sets (10,185 gene sets) from the v.7.4 MSigDB database.[40–42] NCBI Entrez gene identifiers were translated to gene symbols with the web application SynGO.[43] A false discovery rate (FDR) $p < 0.05$ (for all gene set results) was considered significant.[44]

Results

Pneumonia affected individual and control individual characteristics

To identify genetic variants associated with pneumonia susceptibility in African American children, we performed a GWAS of 482 pneumonia affected individuals and up to 2048 control individuals (2,530 total samples), using as affected individuals African American children who were prospectively enrolled in a study of community-acquired pneumonia (Figure 1). Case individuals met the clinical definition of pneumonia including the presence of new infiltrates on chest radiograph. We used as control individuals African American adults enrolled in the University of Michigan MGI biorepository, selecting control individuals who had a similar distribution in the genotype principal component space as the pneumonia affected individuals (Figures S1E, S1F, and S2B) (see subjects and methods). The selected individuals were located between European and African reference samples, indicating minimal admixture from other ancestries (Figure S2B). Affected

Table 1. **Characteristics of pneumonia affected individuals and control individuals in the total sample**

Characteristics	Affected individuals ($n = 482$)	Control individuals ($n = 2,048$)
Female, n (%)	231 (47.9)	1,241 (60.6)
Age (years), median (IQR)	2.2 (1.1–5.4)	51 (39–62)
Comorbid conditions,[a] n (%)		
Asthma	101 (21.0)	543 (26.5)
History of prematurity	55 (11.4)	N/A
Neurological disorders	39 (8.1)	250 (12.2)
Sickle cell disease	59 (12.2)	13 (0.6)
Chronic lung disease	N/A	310 (15.1)
Hospitalized, n (%)	308 (69.9)	N/A
Mechanically ventilated, n (%)	53 (11.0)	N/A
PARDS, n (%)	42 (8.7)	N/A
Death, n (%)	4 (0.8)	N/A

[a]Comorbid conditions in pneumonia affected individuals were determined from the medical chart review with neurological disorders defined as those with seizures or developmental delay. Comorbid conditions in control individuals were defined using ICD-9 codes (see subjects and methods). IQR, interquartile range; N/A, not applicable; PARDS, pediatric acute respiratory distress syndrome.

individual/control individual characteristics are shown in Table 1. The median ages of affected individuals and control individuals were 2.2 (interquartile range [IQR] = 1.1–5.4) and 51 (IQR = 39–62) years of age, respectively.

Genotyping and imputation with TOPMed and 1KG reference panels

All control individuals had been previously genotyped on the CoreExome array, a relatively low coverage array. To maximize the LD coverage for African Americans and to be able to assess potential bias due to differential genotype calling by genotype array, we genotyped all of the 482 pneumonia affected individuals and re-genotyped 518 of the 2,048 control individuals using the much higher coverage MEGA array (genotyping-matched sample) (Figure 1). Using variants present in the total sample (168K array) or the genotyping-matched subset (MEGA array, 835K variants), we imputed allelic dosages using reference panels from either 1KG (2,504 individuals, which contains rs334) or TOPMed (97,256 individuals) (Figure 1), which has higher quality imputation than 1KG but does not have rs334 and other variants in the *HBB* region that were excluded by TOPMed Hardy-Weinberg equilibrium quality control metrics[19,20] (see Table S1). To aid in interpretation of our results, we next sought to understand how the power to detect association differed between the different samples and imputation panels.

Assessment of effective sample size for detection of association signals in the total and genotyping-matched datasets

The power to detect association of a causal imputed variant with pneumonia will be higher when the effective sample size (defined as a sample with equal numbers of affected individuals and control individuals that has the same power as the actual sample) is larger and/or the variant imputation quality is higher.[20] The total sample is larger than the genotyping-matched subset (2,530 vs. 1,000, respectively), but each control beyond the number of affected individuals adds proportionally less information, resulting in a total sample effective sample size of 1,562 and a genotype-matched effective sample size of 999.

When the true genotypes in a study are perfectly imputed (i.e., the directly genotyped and imputed genotypes match exactly), there is no difference in the power to detect association between the imputed and directly genotyped variants. Higher imputation quality is typically observed with larger genotype arrays, larger reference panels, and better reference panel representation of the study samples ancestries.[19,31,45–47] To assess the impact of variant imputation quality on our effective samples size we empirically assessed the imputation quality using 168K and MEGA array genotypes with the TOPMed and 1KG reference panels. We used as gold standard, whole-genome sequenced African American data, and estimated the correlation between the sequenced and the imputed genotype (observed imputation r^2).[31] As reported previously, we found the observed imputation r^2 was lower for variants with lower minor allele frequencies[31] (Figure S3). For SNPs with MAF > 0.01, the average observed r^2 was highest (observed $r^2 = 0.98$) for the TOPMed imputation from the MEGA array and lowest (observed $r^2 = 0.62$) from the 1KG imputation from the 168K array (smaller reference panel and genotyped SNPs) (Figure 2A). To adjust our calculated effective samples sizes for the different imputation qualities, we multiplied the effective sample size by the average imputation quality to

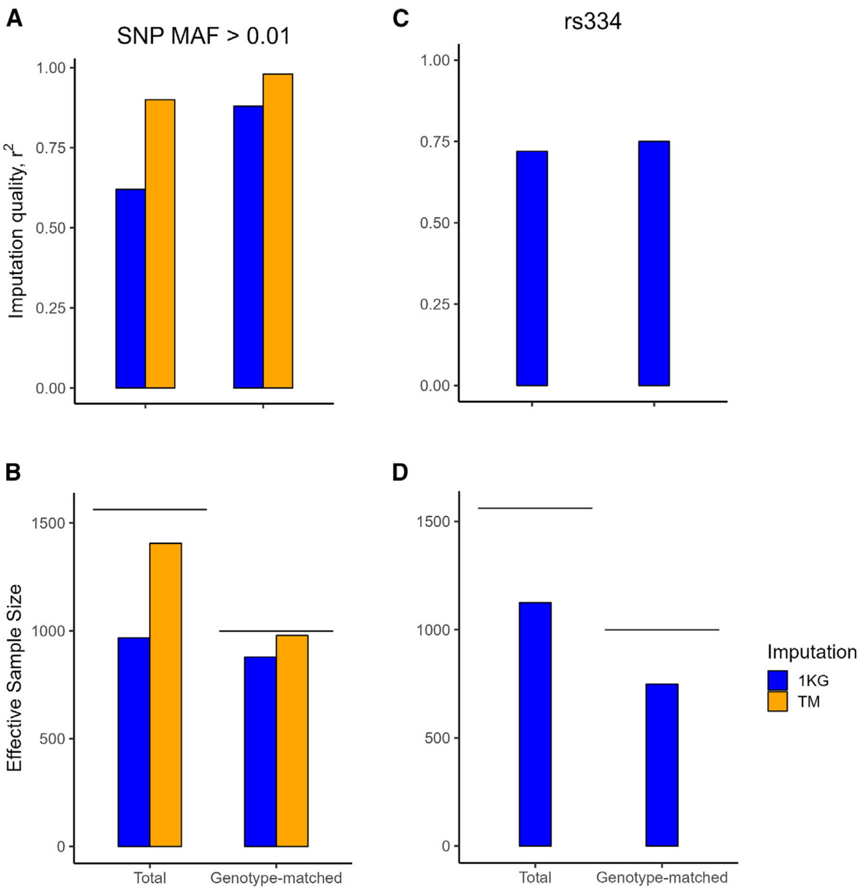

Figure 2. Imputation quality and effective imputation sample size for SNPs with MAF > 0.01 and for rs334
Total and genotype-matched samples bar plots of imputation quality for SNPs with MAF > 0.01(1KG and TOPMed imputation) (A) and rs334 (1KG imputation) (C) Total and genotype-matched samples bar plots of Effective imputation-based sample sizes for SNPs with MAF > 0.01 (B) and rs334 (D). Lines in (B, D) indicate effective sample size assuming perfect genotyping. TM, TOPMed.

give an estimated imputation-based effective sample size ($N_{effective\_imputation}$). For both the 168K and MEGA array data, the TOPMed imputation had a higher effective sample size than the 1KG imputation. The total sample TOPMed $N_{effective\_imputation}$ was 1406, which was substantially higher than the genotyping-matched subset TOPMed $N_{effective\_imputation}$ of 979 (Figure 2B; Table S2). For rs334, the imputation qualities were similar for total and genotype-matched sample 1KG imputation (observed r^2, 0.72 vs 0.75, respectively) (Figure 2C; Table S3); the total sample 1KG rs334 imputation-based effective sample size ($N_{effective\_imputation\ \_rs334}$) was 1,125, which was substantially higher than the genotyping-matched dataset 1KG $N_{effective\_rs334}$ of 749 (Figure 2D; Table S2). Given the higher estimated total sample imputation adjusted effective sample sizes for SNPs with MAF > 0.01 and rs334, we present total sample based-results in the text, and genotyping-matched subset plots in the supplemental information.

Our decision to use the MEGA array rather than the CoreExome array to genotype the genotyping-matched subset decreased the number of variants available for imputation in the total sample from the 188K in the CoreExome array to 168K shared variants from the combination of the CoreExome and MEGA arrays (168K array). The estimated imputation quality was less than 0.05 lower for variants imputed using the 168K array when compared with the 188K CoreExome array for TOPMed and for 1KG based imputation, and thus had minimal effect on our power to detect associations (Figures S3A and S3B).

Pneumonia GWAS

We performed a GWAS of pneumonia using the total sample 1KG and TOPMed imputed dosages and identified significantly associated variants in the β-globin locus on chromosome 11 ($p < 5 \times 10^{-8}$) (Figures 3A, 3B, and S4). In the 1KG imputation-based analysis, the most significantly associated variant was rs334, a nonsynonymous SNP in the β-globin gene, for which the A allele in homozygous or compound heterozygous form is the most common cause of SCD (odds ratio [OR] = 2.76 for allele A, $p = 5.9 \times 10^{-19}$, affected individual and control individual frequencies of 15.7% and 6.2%, respectively) (Tables 2 and S4; Figure 4A). In the TOPMed imputation-based analysis, the most significantly associated variant was rs2226952, located in the β-globin locus control region (OR = 2.1 for G allele, $p = 5.1 \times 10^{-16}$, affected individual and control individual allele frequencies of 24.0% and 13.1%, respectively (Tables 2, 4B, and S4). rs2226952 was also associated with pneumonia in the 1KG imputed data (OR = 2.29, $p = 2.0 \times 10^{-15}$) and is in moderate LD with rs334 in the total sample (Figure 4A; Table S5).

To confirm that the chromosome 11 results for the total sample were not due to differences in affected individual and control individual genotyping between the MEGA (482 affected individuals and 518 control individuals) and the CoreExome (1,530 control individuals) arrays, we restricted our analysis to the genotyping-matched samples (MEGA array). Consistent with the total dataset results, we only observed genome-wide significant variants in the β-globin locus on chromosome 11 in the 1KG and TOPMed imputation-based analysis (Figure S5). In the 1KG imputation-based analysis, rs334 ($p = 7.1 \times 10^{-10}$) and rs2226952 ($p = 2.2 \times 10^{-10}$) were among the variants

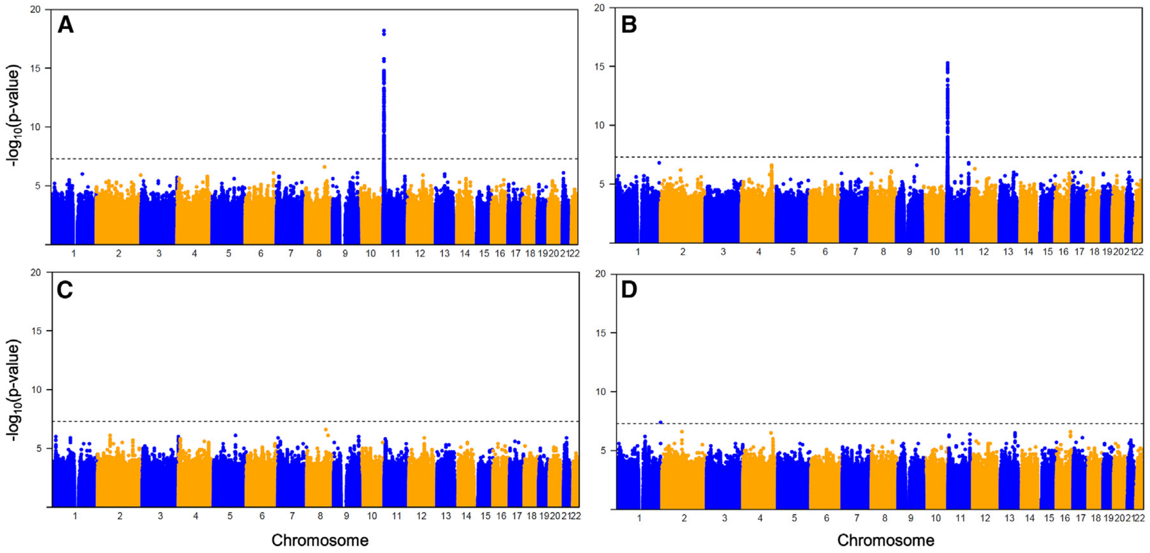

Figure 3. Total sample Manhattan plots of pneumonia GWAS with and without conditioning on rs334
Manhattan plots of total sample imputed with 1KG (A and C) and TOPMed (B and D) reference panels, analyzed using Firth logistic regression for SNPs with MAC ≥ 10. Firth logistic regressions not conditioned (A and B) and conditioned on rs334 (imputed with 1KG) (C and D) dosages. Dotted line, $p = 5 \times 10^{-8}$.

that reached genome-wide significance (Figure S6A). In the TOPMed imputation-based analysis, rs2226952 was the most significant SNP ($p = 5.8 \times 10^{-11}$) (Figure S6B). Thus, the associations of β-globin locus SNPs with pneumonia were detected within and across genotyping arrays.

Fine mapping of the β-globin locus region

To determine if the association of rs334 with pneumonia could account for the association of other variants in the β-globin locus region, we performed conditional logistic regression in the total dataset including rs334 (from 1KG imputation) as a covariate in the analysis. No chromosome 11 variant remained significant after conditioning on rs334 in the 1KG (Figures 3C and 4E; Table 2) or TOPMed (Figures 3D and 4F; Table 2) analysis. The LD r^2 values between rs334 and other pneumonia-associated SNPs were relatively low in the total sample (Figures 4A and 4B), but were somewhat higher in the pneumonia affected individuals, which are enriched for individuals with SCD (Figures 4C and 4D), consistent with positive selection for haplotypes containing the rs334 A allele, which in heterozygous form protects against malaria.[48] Interestingly, the most significant chromosome 11 SNP after conditioning on rs334 (in both the TOPMed and 1KG imputation-based analysis) was rs33930165 (TOPMed OR = 3.89 pre-conditioning and OR = 3.58 post-conditioning with $p = 4.7 \times 10^{-8}$ and $p = 4.7 \times 10^{-7}$, respectively, with risk (T) allele frequencies of 3.5% in affected individuals and 1.0% in control individuals) (Figures 4E and 4F; Table 2). rs33930165 is a nonsynonymous *HBB* SNP in the same codon as rs334; the risk allele, T, changes the amino acid to a lysine resulting in hemoglobin C. Compound heterozygotes of the risk alleles of rs33930165 (T allele) and rs334 (A allele) can cause SCD[16]; rs33930165 TT homozygous individuals have mild anemia, but not SCD.[49] We also conditioned on rs2226952 (imputed with TOPMed or 1KG); rs334 alone remained significant (Figure S7; Table S6).

When conditioning on either rs334 or rs2226952 in the genotyping-matched subset, no other chromosome 11 variant remained genome-wide significant (Figures S6 and S8; Table S7). Thus, in the genotyping-matched subset

Table 2. β-Globin locus SNPs most significantly associated with pneumonia in the total sample without and with conditioning on rs334

		1KG reference panel				TOPMed reference panel			
		Unconditioned		Conditioned on rs334		Unconditioned		Conditioned on rs334	
SNP	Risk/ non-risk alleles	OR (95% CI)	*p* value	OR (95% CI)	*p* value	OR (95% CI)	*p* value	OR (95% CI)	*p* value
rs334	A/T	2.76 (2.21–3.46)	5.2×10^{-19}	–	–	N/A	N/A	N/A	N/A
rs33930165	T/C	4.44 (2.52–7.82)	2.2×10^{-7}	4.09 (2.29–7.29)	1.6×10^{-6}	3.89 (2.38–6.35)	4.7×10^{-8}	3.58 (2.18–5.90)	4.7×10^{-7}
rs2226952	G/T	2.29 (1.86–2.81)	2.0×10^{-15}	1.61 (1.26–2.06)	1.2×10^{-4}	2.14 (1.78–2.57)	5.1×10^{-16}	1.61 (1.30–1.99)	1.3×10^{-5}

Variants imputed using 1KG and TOPMed reference panels. Conditional analysis using rs334 (imputed with 1KG reference panel) dosages. The *HBB* gene is transcribed from the reverse strand of the genome (alleles called on the forward strand). SNP, single nucleotide polymorphism; OR, odds ratio; CI, confidence interval; N/A, not applicable as rs334 is not imputed in TOPMed.

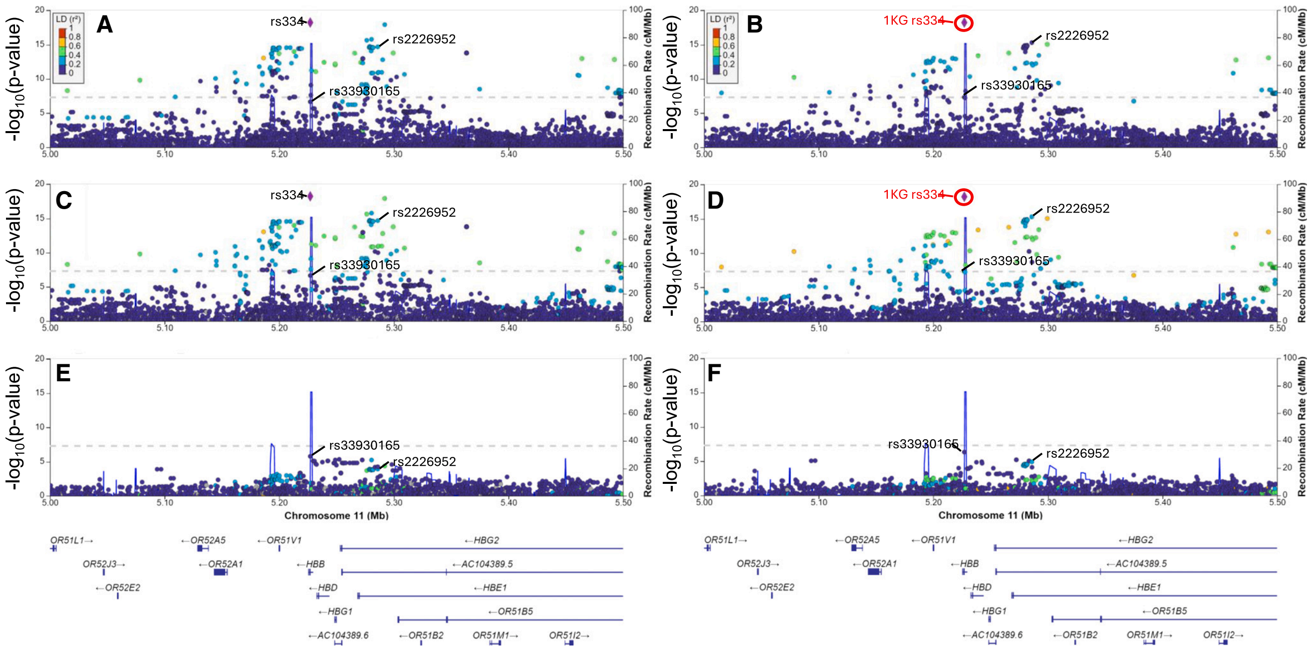

Figure 4. Total sample β-globin locus plots of pneumonia GWAS with and without conditioning on rs334
Plots of β-globin locus for total dataset imputed with 1KG (left) and TOPMed (right). Linkage disequilibrium (LD) to rs334 (1KG imputation) in total sample (A and B). LD to rs334 in affected individuals only (C–F). GWAS analysis conditioned on rs334 dosages (imputed with 1KG) (E and F). All variant and gene positions shown in genome build hg38; 1KG imputed rs334 added to TOPMed plots for comparison purposes (red circle); dotted line, $p = 5 \times 10^{-8}$.

there was no evidence that conditioning on rs334 was more effective than rs2226952 in accounting for other β-globin locus signals, potentially because of the less significant associations signals for both variants in the genotyping-matched subset compared with the total sample or because rs2226952 has a higher observed imputation r^2 than rs334 ($r^2 = 0.97$ compared with 0.75, Table S3).

One chromosome 1 variant, rs12137603, was genome-wide significant in the total sample TOPMed imputed data after conditioning on rs334 (p values without and with conditioning $p = 1.6 \times 10^{-7}$ and 4.23×10^{-8}), but not in the 1KG imputed data (Table S8). This variant is in an intergenic region between the genes for Formin 2 (FMN2) and Gremlin 2 (GREM2) (Figure S9). Interestingly, Gremlin 2 has been reported to be elevated in lung tissue and blood of individuals with idiopathic pulmonary fibrosis and appears to be present in myofibroblasts in fibrotic lung lesions.[50]

Impact of adjustment for SCD on pneumonia association in the β-globin gene region

We next asked if the presence of individuals with SCD could account for the association of pneumonia with rs334 and other variants in the β-globin locus. When we included SCD as a covariate in our logistic analysis, no variant was genome-wide significant (Table 3), suggesting that the association signal we observed in this region is, at least in part, driven by SCD in pneumonia case individuals (Table 3). We tested for the effect of being homozygous for the rs334 risk allele compared with being homozygous for the non-risk allele and observed strong association (OR = 38.8; 95% CI, 14.5–103.8; $p = 2.4 \times 10^{-13}$). In addition, we assessed the effect of being heterozygous for the rs334 A allele by either adjusting for SCD (Table 3) (OR = 1.34; 95% CI, 0.97–1.84; $p = 0.078$) or by removing all individuals with SCD (Table S9) (OR = 1.24; 95% CI, 0.88–1.76; $p = 0.22$); while the ORs for the rs334 A allele were greater than 1, we did not find significant evidence of an increased risk of pneumonia in children who are heterozygous for rs334 A allele, although we have limited power to detect association.

Comparison with previously published pneumonia GWAS

Four published pneumonia GWAS in European or African Ancestry adults have identified nine genome-wide-associated variants.[9,12,13,15] Apart from rs334, in the total sample, we observed nominal evidence of association in the same direction for one variant, rs3131623, located in the HLA class 1 region[9] (OR = 1.49, $p = 0.017$) (Table 4). We did not observe an excess of variants with associations in the same direction between our study and the published studies (six of nine variants had consistent effect directions, $p = 0.17$).

Gene set analysis

To identify biologically related sets of genes that are enriched for pneumonia-associated variants, we performed gene-set analysis of our TOPMed-based total sample association results as described in subjects and methods.

Table 3. Effect of conditioning on sickle cell disease on the association of select β-globin locus variants with pneumonia in the total sample

SNP	Risk/ non-risk alleles	1KG reference panel				TOPMed reference panel			
		Unconditioned		Conditioned on SCD		Unconditioned		Conditioned on SCD	
		OR (95% CI)	*p* value	OR (95% CI)	*p* value	OR (95% CI)	*p* value	OR (95% CI)	*p* value
rs334	A/T	2.76 (2.21–3.46)	5.9×10^{-19}	1.34 (0.97–1.84)	0.078	N/A	N/A	N/A	N/A
rs33930165	T/C	4.44 (2.52–7.82)	2.2×10^{-7}	2.99 (1.60–5.59)	5.5×10^{-4}	3.89 (2.38–6.35)	4.7×10^{-8}	2.82 (1.65–4.83)	1.5×10^{-4}
rs2226952	G/T	2.29 (1.86–2.81)	2.0×10^{-15}	1.61 (1.27–2.03)	5.6×10^{-5}	2.14 (1.78–2.57)	5.1×10^{-16}	1.61 (1.32–1.98)	3.8×10^{-6}

Variants imputed using 1KG and TOPMed reference panels. The *HBB* gene is transcribed from the reverse strand of the genome (alleles called on the forward strand). SNP, single nucleotide polymorphism; SCD, sickle cell disease; OR, odds ratio; CI, confidence interval; N/A, not applicable as rs334 is not imputed in TOPMed.

The GOMF_HEMOGLOBIN_ALPHA_BINDING gene set was significantly positively enriched (TOPMed imputation, beta (SD) = 3.59 (0.70), $p = 3.47 \times 10^{-7}$, FDR $p = 0.0045$); this signal was driven by the association signals in the β-globin locus.

Discussion

We find that variants in the β-globin locus are associated with documented pneumonia in African American children. The most strongly associated variant in the total sample 1KG imputation analysis was rs334, which when homozygous for the A allele, or as a compound heterozygote, causes SCD. When we conditioned on SCD status, all signals in the region (including rs334 and rs2226952), were no longer significant. After conditioning on rs334 in the TOPMed and 1KG imputed data, the most significantly associated variant on chromosome 11 was rs33930165. rs33930165 is in the same codon as rs334 (but not in LD with it) and the compound heterozygote of the rs33930165 T allele and the rs334 A allele causes SCD. Thus, it seems plausible that both rs334 and rs33930165 contribute to the risk of pneumonia through increased risk of SCD. We did not find evidence of an increased risk of pneumonia in children who are heterozygous for rs334 A allele but do not have SCD (children with sickle cell trait) although we have limited power to detect weaker associations.

Our findings are consistent with epidemiologic studies showing an increased risk of pneumonia for children with SCD.[17] They are also consistent with the association of rs334 with pneumonia defined by diagnosis code in adult African American individuals in the BioVue cohort.[13] Interestingly rs334 was not associated with self-reported childhood or adult pneumonia in African American individuals in the COPDgene study.[14] This could be because of uncertainties in self-reported pneumonia, or potential disenrichment for individuals with SCD due to the requirement for smoking >10 pack years and older age of study participants. Apart from rs344, we observed no significant increased risk of pneumonia in African American children with any of the variants reported to be associated with pneumonia in adults of European or African Ancestry,[9,12,13,15] although we saw nominal evidence of association in the same direction for rs3131623, in the HLA class 1 region.[9]

The most strongly associated variant in the TOPMed imputation analysis, rs2226952, resides within the β-globin locus control region, an area involved in controlling expression of five developmentally regulated β-like globin genes[51–53] including the fetal hemoglobin genes, whose levels of expression affect the severity of SCD.[54,55] Individuals with lower levels of fetal hemoglobin have, on average, more severe SCD[54,55] and a higher risk of pneumonia[55] and acute chest syndrome.[56] However, in our sample the previously reported hemoglobin F-associated β-globin locus SNP, rs10128556,[57] is in low LD (r^2) with rs2226952 ($r^2 = 0.0258$, D' = 0.931) and is not associated with pneumonia at genome-wide levels ($p = 0.04$). rs334 is not present in the TOPMed version r2 reference panel because it was out of Hardy-Weinberg equilibrium likely due to the shorter life expectancy in people with SCD (individuals homozygous for the A allele) and/or due to potential exclusion from the participating studies. rs344 is only moderately well imputed using the 1KG reference panel. It is not clear if rs2226952 or another variant(s) independently affects the risk of pneumonia. Overall, the removal of reference panel variants out of Hardy-Weinberg equilibrium because of poor genotyping quality can increase the quality of the panel, but care has to be taken to retain variants with strong effects on mortality and those in LD with them. Imputation with future large reference panels that include rs334 and substantial numbers of African ancestry individuals will help clarify this issue.

Because the pneumonia affected individuals were collected without matching control individuals, we performed our affected individual/control individual comparison using existing MGI study control individuals which had been previously genotyped using CoreExome array. We chose to regenotype a subset of the MGI control individuals along with the affected individuals in order to identify and remove variants that differed by more than one genotype in control individuals thereby reducing the likelihood of artifactual findings due to the use of different arrays for genotyping the affected individuals and control

Table 4. Total sample results for published pneumonia GWAS variants

Published pneumonia GWAS signals										Current total sample GWAS[a]			
Study	First author	Anc	Affected individuals *N*/control individuals *N*[b]	Gene region	SNP	Effect/ non-effect alleles	Effect allele freq	OR/effect direction[c]	*p* value	Effect allele freq	OR/effect direction	*p* value	Consistent effect direction[d]
23andMe	Tian[9]	EA	41K/90K	*HLA* class I region	rs3131623	T/A	0.85	1.1	1.99×10^{-15}	0.93	1.47	0.017	yes
BioVue	Chen[13]	EA	9K/61K	*CFTR*	rs113827944	A/G	0.021	1.84	1.84×10^{-36}	0.004	0.33	0.22	no
BioVue	Chen[13]	AA	2K/14K	*HBB*	rs334	A/T	0.058	1.63	3.5×10^{-13}	0.08	2.76	5.9×10^{-19}	yes
UK Biobank & FinnGen	Campos[12]	EA	25K/526K	NR	rs11708673	A/T	NR	neg effect	4.21×10^{-8}	0.11	1.01	0.96	no
23andMe & FinnGen	Reay[15]	EA	74K/317K	*MHC*	rs9275211	C/T	0.18[e] 0.16[f]	1.06	3.83×10^{-14}	0.14	1.08	0.45	yes
23andMe & FinnGen	Reay[15]	EA	74K/317K	upstream of *MUC5AC*	rs11245979	C/T	0.31[e] 0.38[f]	1.05	7.25×10^{-11}	0.75	1.11	0.26	yes
23andMe & FinnGen	Reay[15]	EA	74K/317K	*TNFRSF1A*	rs4149581	C/T	0.42[e] 0.42[f]	0.96	3.22×10^{-9}	0.13	0.89	0.34	yes
23andMe & FinnGen	Reay[15]	EA	74K/317K	near *PTGER4*	rs9283753	T/C	0.57[e] 0.50[f]	1.04	3.39×10^{-9}	0.58	1.07	0.34	yes
23andMe & FinnGen	Reay[15]	EA	74K/317K	*IL6R*	rs6684439	T/C	0.37[e] 0.30[f]	1.04	3.05×10^{-8}	0.36	0.94	0.42	no

Anc, ancestry; SNP, single nucleotide polymorphism; freq, frequency; OR, odds ratio; EA, European Ancestry; AA, African Ancestry; NR, not reported; neg, negative.
[a]TOPMed imputation for all variants except rs334; 1KG imputation for rs334.
[b]Affected individuals and control individuals *N* values rounded to 1,000s.
[c]Using the published study effect allele.
[d]Consistency of effect directions between the published study and in our GWAS (yes, same effect direction; no, opposite effect direction).
[e]Non-Finnish European ancestry effect allele frequency.
[f]Finnish European ancestry effect allele frequency.

individuals. We chose to genotype samples using the MEGA array which has more variants than the CoreExome array and was designed to improve imputation in non-European samples.[58] This strategy affected our analysis in two main ways. First, for the total sample we had a smaller set of variants in common between the two arrays (168K) than we would have had if we had used the CoreExome array (188K) to genotype the affected individuals and matched control individuals. However, the imputation quality was only slightly less than it would have been if we had chosen to genotype the affected individuals on the CoreExome array. Second, our strategy resulted in a smaller genotype-matched sample (n = 1,000) imputed from 835K variants which had higher imputation quality than the total sample. To assess the relative power of the total and the genotype-matched samples, we estimated the effective sample size scaled by the average observed imputation quality r^2. We also used this strategy to assess the $N_{effective\_imputation\_SNP}$ for the rs334 variant. The imputation quality adjusted-effective samples sizes informed our decision to present results from the total sample in the main text and to use the genotype-matched sample to confirm results.

This study has several potential limitations. First, our affected individuals are children from four communities and our control individuals are adults undergoing elective surgery at the University of Michigan. However, we selected the affected individuals and control individuals to have similar genetic ancestry distributions, and we corrected for genetic ancestry using genetic principal components. Second, because of the difficulty in ascertaining which adults had a previous diagnosis of pneumonia using billing codes, we did not assess pneumonia in the control individuals. This likely reduced the power to detect variants associated with pneumonia, especially variants with a weaker association. The adult control individuals also had a higher rate of asthma and chronic lung disease than reported in the general population.[59,60] This may be because control individuals were recruited from adults undergoing elective surgery or diagnostic procedures which may have enriched for individuals with pre-existing conditions. Third, the difference we see between childhood affected individuals and adult control individuals could arise from a higher mortality rate from SCD as individuals age. In newborn African American children, the prevalence of SCD trait (individuals with a single SCD risk allele), is estimated at 1 in 13 (7.6%) and of SCD is estimated at 1 in 365 (0.27%) (https://www.cdc.gov/ncbddd/sicklecell/data.html). Within the pneumonia affected individuals, 59 of 482 pneumonia affected individuals (12%) had SCD, and among the adult control individuals 13 of 2,048 (0.6%) had SCD, thus pneumonia affected individuals were greatly enriched in children with SCD compared with the expected number in children. Fourth, our Emergency Department and hospital-based case samples may have been enriched for children with SCD, due to concerns about serious illness in children with SCD and pneumonia. However, our results are consistent with GWAS results seen for African American adults with electronic health record-identified pneumonia.[13] Fifth, approximately three-fourths of our control individuals were genotyped on a different array than affected individuals. To reduce bias, we imputed variants for the total sample set using a highly concordant set of SNPs present on both the MEGA and CoreExome arrays. We observed the same β-globin locus signal in the genotyping-matched subset. Lastly, we have a relatively small number of affected individuals (n = 482) and there are almost certainly other smaller effect size SNPs associated with pneumonia.

In summary, homozygous or compound heterozygous SCD risk alleles for rs334 and rs33930165 are associated with increased risk of pneumonia, indicating that most, if not all, of the risk from this locus is due to the increased susceptibility to pneumonia caused by SCD. These findings suggest that being homozygous for the SCD risk variant rs334 is the largest single variant genetic contributor to pneumonia risk in African American children and adults.

Data and code availability

The datasets supporting the current study have not been deposited in a data repository because the genotype data cannot be shared. The pneumonia case data consents do not include sharing of data. The Michigan Genomics Initiative data is regulated by Michigan Medicine, which does not allow sharing of the data. We have provided our GWAS summary statistics to the NHGRI-EBI GWAS Catalog for access (https://www.ebi.ac.uk/gwas/). The accession numbers for the data reported in this paper are GWAS Catalog: GCST90446235, GCST90446236, GCST90446237, and GCST90446238.

Acknowledgments

The authors acknowledge the Michigan Genomics Initiative participants, Precision Health at the University of Michigan, the University of Michigan Medical School Data Office for Clinical and Translational Research, the University of Michigan Medical School Central Biorepository, and the University of Michigan Advanced Genomics Core for providing data and specimen storage, management, processing, and distribution services in support of the research reported in this publication. This research was also supported in part through computational resources and services provided by Advanced Research Computing (ARC), a division of Information and Technology Services (ITS) at the University of Michigan, Ann Arbor. This research received funding from the Department of Pediatrics, University of Michigan Medical School and the University of Michigan (to M.K.D.) and from R01 HG009976.

Author contributions

Study design: M.K.D., L.J.S., M.W.Q; Data acquisition: M.K.D., S.Z., N.L.N.H, M.W.Q.; Analysis and interpretation: M.K.D., L.J.S, N.L.N.H, S.C.H., K.M. T.M.; Writing: M.K.D., L.J.S, N.L.N.H. All authors participated in critical revision of the paper and approved submission of the manuscript.

Declaration of interests

The authors declare no competing interests.

Supplemental information

Supplemental information can be found online at https://doi.org/10.1016/j.xhgg.2024.100374.

Web resources

NCBI dbSNP: https://www.ncbi.nlm.nih.gov/snp/rs334

OMIM, An Online Catalog of Human Genes and Genetic Disorders: https://www.omim.org/

Michigan Genomics Initiative: https://precisionhealth.umich.edu/our-research/michigangenomics/

ICD9Data.com: http://www.icd9data.com/

The PAGE Study, Population Architecture using Genomics and Epidemiology: www.pagestudy.org and https://www.pagestudy.org/index.php/multi-ethnic-genotyping-array

PLINK 1.9 beta: www.cog-genomics.org/plink/1.9/

McCarthy Group Tools: https://www.chg.ox.ac.uk/~wrayner/tools/index.html#Checking

TOPMed Imputation Server: https://imputation.biodatacatalyst.nhlbi.nih.gov/#!

Michigan Imputation Server 2: https://imputationserver.sph.umich.edu/index.html#!

KING: Kinship-based INference for Gwas: https://www.kingrelatedness.com/

University of Michigan Center for Statistical Genetics LASER Service: https://laser.sph.umich.edu/index.html

PLINK 2.0 alpha: www.cog-genomics.org/plink/2.0/

LocusZoom: https://my.locuszoom.org/

LocalZoom: Plot your own data with LocusZoom.js: https://abought.github.io/localzoom/

GWASTutorial: https://cloufield.github.io/GWASTutorial/09_Gene_based_analysis/

MSigDB, Molecular Signatures Database: https://www.gsea-msigdb.org/gsea/msigdb

Received: March 29, 2024
Accepted: October 14, 2024

References

1. Liu, L., Oza, S., Hogan, D., Chu, Y., Perin, J., Zhu, J., Lawn, J.E., Cousens, S., Mathers, C., and Black, R.E. (2016). Global, regional, and national causes of under-5 mortality in 2000-15: an updated systematic analysis with implications for the Sustainable Development Goals. Lancet *388*, 3027–3035.
2. McAllister, D.A., Liu, L., Shi, T., Chu, Y., Reed, C., Burrows, J., Adeloye, D., Rudan, I., Black, R.E., Campbell, H., and Nair, H. (2019). Global, regional, and national estimates of pneumonia morbidity and mortality in children younger than 5 years between 2000 and 2015: a systematic analysis. Lancet Global Health *7*, e47–e57.
3. McDermott, K.I.W.H., and Roemer, M.A. (2021). Most Frequent Principal Diagnoses for Inpatients Stays in US Hospitals, 2018 (HCUP Statistical Brief #277).
4. National Center for Health Statistics. Health, U.S. (2019). Table 7. Leading Causes of Death and Numbers of Deaths, by Age: United States, 1980 and 2018 (Centers for Disease Control and Prevention). https://www.cdc.gov/nchs/data/hus/2019/007-508.pdf.
5. Jain, S., Williams, D.J., Arnold, S.R., Ampofo, K., Bramley, A.M., Reed, C., Stockmann, C., Anderson, E.J., Grijalva, C.G., Self, W.H., et al. (2015). Community-acquired pneumonia requiring hospitalization among U.S. children. N. Engl. J. Med. *372*, 835–845.
6. Dela Cruz, C.S., Wunderink, R.G., Christiani, D.C., Cormier, S.A., Crothers, K., Doerschuk, C.M., Evans, S.E., Goldstein, D.R., Khatri, P., Kobzik, L., et al. (2018). Future Research Directions in Pneumonia. NHLBI Working Group Report. Am. J. Respir. Crit. Care Med. *198*, 256–263.
7. Sørensen, T.I., Nielsen, G.G., Andersen, P.K., and Teasdale, T.W. (1988). Genetic and environmental influences on premature death in adult adoptees. N. Engl. J. Med. *318*, 727–732.
8. Chapman, S.J., and Hill, A.V.S. (2012). Human genetic susceptibility to infectious disease. Nat. Rev. Genet. *13*, 175–188.
9. Tian, C., Hromatka, B.S., Kiefer, A.K., Eriksson, N., Noble, S.M., Tung, J.Y., and Hinds, D.A. (2017). Genome-wide association and HLA region fine-mapping studies identify susceptibility loci for multiple common infections. Nat. Commun. *8*, 599.
10. Pairo-Castineira, E., Clohisey, S., Klaric, L., Bretherick, A.D., Rawlik, K., Pasko, D., Walker, S., Parkinson, N., Fourman, M.H., Russell, C.D., et al. (2021). Genetic mechanisms of critical illness in COVID-19. Nature *591*, 92–98.
11. Severe Covid-19 GWAS Group, Ellinghaus, D., Degenhardt, F., Bujanda, L., Buti, M., Albillos, A., Invernizzi, P., Fernández, J., Prati, D., Baselli, G., et al. (2020). Genomewide Association Study of Severe Covid-19 with Respiratory Failure. N. Engl. J. Med. *383*, 1522–1534.
12. Campos, A.I., Kho, P., Vazquez-Prada, K.X., García-Marín, L.M., Martin, N.G., Cuéllar-Partida, G., and Rentería, M.E. (2021). Genetic Susceptibility to Pneumonia: A GWAS Meta-Analysis Between the UK Biobank and FinnGen. Twin Res. Hum. Genet. *24*, 145–154.
13. Chen, H.H., Shaw, D.M., Petty, L.E., Graff, M., Bohlender, R.J., Polikowsky, H.G., Zhong, X., Kim, D., Buchanan, V.L., Preuss, M.H., et al. (2021). Host genetic effects in pneumonia. Am. J. Hum. Genet. *108*, 194–201.
14. Hayden, L.P., Cho, M.H., McDonald, M.L.N., Crapo, J.D., Beaty, T.H., Silverman, E.K., Hersh, C.P.; and COPDGene Investigators * (2017). Susceptibility to Childhood Pneumonia: A Genome-Wide Analysis. Am. J. Respir. Cell Mol. Biol. *56*, 20–28.
15. Reay, W.R., Geaghan, M.P., 23andMe Research Team, and Cairns, M.J. (2022). The genetic architecture of pneumonia susceptibility implicates mucin biology and a relationship with psychiatric illness. Nat. Commun. *13*, 3756.
16. Piel, F.B., Steinberg, M.H., and Rees, D.C. (2017). Sickle Cell Disease. N. Engl. J. Med. *377*, 305.
17. Ochocinski, D., Dalal, M., Black, L.V., Carr, S., Lew, J., Sullivan, K., and Kissoon, N. (2020). Life-Threatening Infectious Complications in Sickle Cell Disease: A Concise Narrative Review. Front. Pediatr. *8*, 38.
18. Sirugo, G., Williams, S.M., and Tishkoff, S.A. (2019). The Missing Diversity in Human Genetic Studies. Cell *177*, 1080.
19. 1000 Genomes Project Consortium, Auton, A., Brooks, L.D., Durbin, R.M., Garrison, E.P., Kang, H.M., Korbel, J.O., Marchini, J.L., McCarthy, S., McVean, G.A., and Abecasis, G.R. (2015). A global reference for human genetic variation. Nature *526*, 68–74.

20. Taliun, D., Harris, D.N., Kessler, M.D., Carlson, J., Szpiech, Z.A., Torres, R., Taliun, S.A.G., Corvelo, A., Gogarten, S.M., Kang, H.M., et al. (2021). Sequencing of 53,831 diverse genomes from the NHLBI TOPMed Program. Nature *590*, 290–299.
21. Little, J., Higgins, J.P.T., Ioannidis, J.P.A., Moher, D., Gagnon, F., von Elm, E., Khoury, M.J., Cohen, B., Davey-Smith, G., Grimshaw, J., et al. (2009). Strengthening the reporting of genetic association studies (STREGA): an extension of the STROBE Statement. Hum. Genet. *125*, 131–151.
22. Baughn, J.M., Quasney, M.W., Simpson, P., Merchant, D., Li, S.H., Levy, H., and Dahmer, M.K. (2012). Association of cystic fibrosis transmembrane conductance regulator gene variants with acute lung injury in African American children with pneumonia. Crit. Care Med. *40*, 3042–3049.
23. Perez-Marques, F., Simpson, P., Yan, K., Quasney, M.W., Halligan, N., Merchant, D., and Dahmer, M.K. (2016). Association of polymorphisms in genes of factors involved in regulation of splicing of cystic fibrosis transmembrane conductance regulator mRNA with acute respiratory distress syndrome in children with pneumonia. Crit. Care *20*, 281.
24. Fritsche, L.G., Gruber, S.B., Wu, Z., Schmidt, E.M., Zawistowski, M., Moser, S.E., Blanc, V.M., Brummett, C.M., Kheterpal, S., Abecasis, G.R., and Mukherjee, B. (2018). Association of Polygenic Risk Scores for Multiple Cancers in a Phenome-wide Study: Results from The Michigan Genomics Initiative. Am. J. Hum. Genet. *102*, 1048–1061.
25. Wojcik, G.L., Graff, M., Nishimura, K.K., Tao, R., Haessler, J., Gignoux, C.R., Highland, H.M., Patel, Y.M., Sorokin, E.P., Avery, C.L., et al. (2019). Genetic analyses of diverse populations improves discovery for complex traits. Nature *570*, 514–518.
26. Chang, C.C., Chow, C.C., Tellier, L.C., Vattikuti, S., Purcell, S.M., and Lee, J.J. (2015). Second-generation PLINK: rising to the challenge of larger and richer datasets. GigaScience *4*, 7.
27. Johnson, E.O., Hancock, D.B., Levy, J.L., Gaddis, N.C., Saccone, N.L., Bierut, L.J., and Page, G.P. (2013). Imputation across genotyping arrays for genome-wide association studies: assessment of bias and a correction strategy. Hum. Genet. *132*, 509–522.
28. Das, S., Forer, L., Schönherr, S., Sidore, C., Locke, A.E., Kwong, A., Vrieze, S.I., Chew, E.Y., Levy, S., McGue, M., et al. (2016). Next-generation genotype imputation service and methods. Nat. Genet. *48*, 1284–1287.
29. Loh, P.R., Danecek, P., Palamara, P.F., Fuchsberger, C., A Reshef, Y., K Finucane, H., Schoenherr, S., Forer, L., McCarthy, S., Abecasis, G.R., et al. (2016). Reference-based phasing using the Haplotype Reference Consortium panel. Nat. Genet. *48*, 1443–1448.
30. Fuchsberger, C., Abecasis, G.R., and Hinds, D.A. (2015). minimac2: faster genotype imputation. Bioinformatics *31*, 782–784.
31. Hanks, S.C., Forer, L., Schönherr, S., LeFaive, J., Martins, T., Welch, R., Gagliano Taliun, S.A., Braff, D., Johnsen, J.M., Kenny, E.E., et al. (2022). Extent to which array genotyping and imputation with large reference panels approximate deep whole-genome sequencing. Am. J. Hum. Genet. *109*, 1653–1666.
32. Manichaikul, A., Mychaleckyj, J.C., Rich, S.S., Daly, K., Sale, M., and Chen, W.M. (2010). Robust relationship inference in genome-wide association studies. Bioinformatics *26*, 2867–2873.
33. Price, A.L., Weale, M.E., Patterson, N., Myers, S.R., Need, A.C., Shianna, K.V., Ge, D., Rotter, J.I., Torres, E., Taylor, K.D., et al. (2008). Long-range LD can confound genome scans in admixed populations. Am. J. Hum. Genet. *83*, 132–139. author reply 135-139.
34. Wang, C., Zhan, X., Liang, L., Abecasis, G.R., and Lin, X. (2015). Improved ancestry estimation for both genotyping and sequencing data using projection procrustes analysis and genotype imputation. Am. J. Hum. Genet. *96*, 926–937.
35. Wang, M., Yu, Y., Haberer, G., Marri, P.R., Fan, C., Goicoechea, J.L., Zuccolo, A., Song, X., Kudrna, D., Ammiraju, J.S.S., et al. (2014). The genome sequence of African rice (Oryza glaberrima) and evidence for independent domestication. Nat. Genet. *46*, 982–988.
36. Firth, D. (1993). Bias reduction of maximum likelihood estimates. Biometrika *80*, 27–38.
37. Price, A.L., Patterson, N.J., Plenge, R.M., Weinblatt, M.E., Shadick, N.A., and Reich, D. (2006). Principal components analysis corrects for stratification in genome-wide association studies. Nat. Genet. *38*, 904–909.
38. Boughton, A.P., Welch, R.P., Flickinger, M., VandeHaar, P., Taliun, D., Abecasis, G.R., and Boehnke, M. (2021). LocusZoom.js: Interactive and embeddable visualization of genetic association study results. Bioinformatics *37*, 3017–3018.
39. de Leeuw, C.A., Neale, B.M., Heskes, T., and Posthuma, D. (2016). The statistical properties of gene-set analysis. Nat. Rev. Genet. *17*, 353–364.
40. Subramanian, A., Tamayo, P., Mootha, V.K., Mukherjee, S., Ebert, B.L., Gillette, M.A., Paulovich, A., Pomeroy, S.L., Golub, T.R., Lander, E.S., and Mesirov, J.P. (2005). Gene set enrichment analysis: a knowledge-based approach for interpreting genome-wide expression profiles. Proc. Natl. Acad. Sci. USA *102*, 15545–15550.
41. Liberzon, A., Birger, C., Thorvaldsdóttir, H., Ghandi, M., Mesirov, J.P., and Tamayo, P. (2015). The Molecular Signatures Database (MSigDB) hallmark gene set collection. Cell Syst. *1*, 417–425.
42. Liberzon, A., Subramanian, A., Pinchback, R., Thorvaldsdóttir, H., Tamayo, P., and Mesirov, J.P. (2011). Molecular signatures database (MSigDB) 3.0. Bioinformatics *27*, 1739–1740.
43. Koopmans, F., van Nierop, P., Andres-Alonso, M., Byrnes, A., Cijsouw, T., Coba, M.P., Cornelisse, L.N., Farrell, R.J., Goldschmidt, H.L., Howrigan, D.P., et al. (2019). SynGO: An Evidence-Based, Expert-Curated Knowledge Base for the Synapse. Neuron *103*, 217–234.e4.
44. Benjamini, Y., and Hochberg, Y. (1995). Controlling the false discovery rate: A practical and powerful approach to multiple testing. J. Roy. Stat. Soc. B *57*, 289–300.
45. Das, S., Abecasis, G.R., and Browning, B.L. (2018). Genotype Imputation from Large Reference Panels. Annu. Rev. Genom. Hum. Genet. *19*, 73–96.
46. McCarthy, S., Das, S., Kretzschmar, W., Delaneau, O., Wood, A.R., Teumer, A., Kang, H.M., Fuchsberger, C., Danecek, P., Sharp, K., et al. (2016). A reference panel of 64,976 haplotypes for genotype imputation. Nat. Genet. *48*, 1279–1283.
47. Kowalski, M.H., Qian, H., Hou, Z., Rosen, J.D., Tapia, A.L., Shan, Y., Jain, D., Argos, M., Arnett, D.K., Avery, C., et al. (2019). Use of >100,000 NHLBI Trans-Omics for Precision Medicine (TOPMed) Consortium whole genome sequences improves imputation quality and detection of rare variant associations in admixed African and Hispanic/Latino populations. PLoS Genet. *15*, e1008500.
48. Piel, F.B., Patil, A.P., Howes, R.E., Nyangiri, O.A., Gething, P.W., Williams, T.N., Weatherall, D.J., and Hay, S.I. (2010).

Global distribution of the sickle cell gene and geographical confirmation of the malaria hypothesis. Nat. Commun. *1*, 104.
49. Piel, F.B., Adamkiewicz, T.V., Amendah, D., Williams, T.N., Gupta, S., and Grosse, S.D. (2016). Observed and expected frequencies of structural hemoglobin variants in newborn screening surveys in Africa and the Middle East: deviations from Hardy-Weinberg equilibrium. Genet. Med. *18*, 265–274.
50. Huan, C., Xu, W., Liu, Y., Ruan, K., Shi, Y., Cheng, H., Zhang, X., Ke, Y., and Zhou, J. (2021). Gremlin2 Activates Fibroblasts to Promote Pulmonary Fibrosis Through the Bone Morphogenic Protein Pathway. Front. Mol. Biosci. *8*, 683267.
51. Vinjamur, D.S., Bauer, D.E., and Orkin, S.H. (2018). Recent progress in understanding and manipulating haemoglobin switching for the haemoglobinopathies. Br. J. Haematol. *180*, 630–643.
52. Orkin, S.H., and Bauer, D.E. (2019). Emerging Genetic Therapy for Sickle Cell Disease. Annu. Rev. Med. *70*, 257–271.
53. Shen, Y., Verboon, J.M., Zhang, Y., Liu, N., Kim, Y.J., Marglous, S., Nandakumar, S.K., Voit, R.A., Fiorini, C., Ejaz, A., et al. (2021). A unified model of human hemoglobin switching through single-cell genome editing. Nat. Commun. *12*, 4991.
54. Platt, O.S., Brambilla, D.J., Rosse, W.F., Milner, P.F., Castro, O., Steinberg, M.H., and Klug, P.P. (1994). Mortality in sickle cell disease. Life expectancy and risk factors for early death. N. Engl. J. Med. *330*, 1639–1644.
55. Castro, O., Brambilla, D.J., Thorington, B., Reindorf, C.A., Scott, R.B., Gillette, P., Vera, J.C., and Levy, P.S. (1994). The acute chest syndrome in sickle cell disease: incidence and risk factors. The Cooperative Study of Sickle Cell Disease. Blood *84*, 643–649.
56. Pincez, T., Lo, K.S., D'Orengiani, A., Garrett, M.E., Brugnara, C., Ashley-Koch, A.E., Telen, M.J., Galacteros, F., Joly, P., Bartolucci, P., et al. (2022). Variation and impact of polygenic hematological traits in monogenic sickle cell disease. Haematologica *108*, 870–881.
57. Galarneau, G., Palmer, C.D., Sankaran, V.G., Orkin, S.H., Hirschhorn, J.N., and Lettre, G. (2010). Fine-mapping at three loci known to affect fetal hemoglobin levels explains additional genetic variation. Nat. Genet. *42*, 1049–1051.
58. Bien, S.A., Wojcik, G.L., Zubair, N., Gignoux, C.R., Martin, A.R., Kocarnik, J.M., Martin, L.W., Buyske, S., Haessler, J., Walker, R.W., et al. (2016). Strategies for Enriching Variant Coverage in Candidate Disease Loci on a Multiethnic Genotyping Array. PLoS One *11*, e0167758.
59. Pate, C.A., Zahran, H.S., Qin, X., Johnson, C., Hummelman, E., and Malilay, J. (2021). Asthma Surveillance - United States, 2006-2018. MMWR. Surveill. Summ. *70*, 1–32.
60. Liu, Y., Carlson, S.A., Watson, K.B., Xu, F., and Greenlund, K.J. (2023). Trends in the Prevalence of Chronic Obstructive Pulmonary Disease Among Adults Aged >/=18 Years - United States, 2011-2021. MMWR Morb. Mortal. Wkly. Rep. *72*, 1250–1256.

HGGA, Volume *6*

Supplemental information

Variants in the β-globin locus are associated with pneumonia in African American children

Nadine L.N. Halligan, Sarah C. Hanks, Karen Matsuo, Taylor Martins, Sebastian Zöllner, Michael W. Quasney, Laura J. Scott, and Mary K. Dahmer

Supplemental Information Table of Contents

Table S1. Number of Variants used for Imputation and Obtained from Imputation of Pneumonia Case/control Dataset and Imputation Reference Panel.

Figure S1. Genotype Principal Component Analysis (PCA) from PLINK and Genetic Ancestry PCA from LASER/TRACE of Total Sample before and after Removing Samples with PC1 < -0.025.

Figure S2. Genetic Ancestry Principal Components from LASER/TRACE Analysis in the Total Sample and Human Genome Diversity Project Samples.

Figure S3. Observed Imputation Quality of InPSYght Genotypes.

Table S2. Average Effective Sample Size Adjusted for Imputation.

Table S3. Estimated and Observed Imputation Quality of β-globin SNPs Imputed with 1KG or TOPMed Reference Panels.

Figure S4. Total Sample Pneumonia GWAS QQ Plots.

Table S4. Total Sample β-globin Locus SNP Risk Allele Frequencies by Pneumonia Case/control Status and Imputation Reference Panel.

Table S5. Linkage Disequilibrium Between Selected SNPs in the Total Sample and Cases only.

Figure S5. Genotyping-matched Subset Pneumonia GWAS Manhattan and QQ Plots by Imputation Reference Panel.

Figure S6. Genotyping-matched Subset β-globin Locus Plots of Plots of Pneumonia GWAS Results with and without Conditioning on 1KG rs334 dosages.

Figure S7. Total Sample β-globin Locus Plots of Pneumonia GWAS Results with and without Conditioning on rs2226952 Dosages.

Table S6. Total Pneumonia GWAS Results for Selected β-globin Locus SNPs with and without Conditioning on rs2226952.

Figure S8. Genotyping-matched Subset β-globin Locus Plots of Pneumonia GWAS Results with and without Conditioning on 1KG rs2226952 Dosages.

Table S7. Genotyping-matched Subset Pneumonia GWAS Results for Selected β-globin SNPs with and without Conditioning with rs334 and rs2226952.

Table S8. TOPMed Imputed Total Sample Pneumonia GWAS Genome-wide Significant SNP after Conditioning with 1KG rs334 Dosages.

Figure S9. Total Sample Locus Plot of GWAS Genome-wide Significant SNP after Conditioning on 1KG rs334 Dosages.

Table S9. Pneumonia GWAS Results for Selected β-globin SNPs with and without Individuals with Sickle Cell Disease.

Table S1. Number of Variants used for Imputation and Obtained from Imputation by Pneumonia Case/control Dataset and Imputation Reference Panel

Dataset		Imputation Information			Imputed SNPs, N			Imputed SNPs with MAC≥ 10, N	
**Name**	**Array**	**SNPs Used, N**	**Samples, N**	**Reference Panel**	**Total**	**MAC < 10**	**MAC ≥ 10**	**MAF ≤ 0.01**	**MAF > 0.01**
Total	168K combined	168,220	2,530	TOPMed	65,908,902	42,047,233	23,861,669	8,566,724	15,294,945
Total	168K combined	168,220	2,530	1KG	24,228,518	4,589,186	19,639,332	5,565,104	14,074,228
Genotyping-matched subset	MEGA	834,828	1,000	TOPMed	65,908,902	47,271,085	18,637,817	3,324,520	15,313,297
Genotyping-matched subset	MEGA	834,828	1,000	1KG	24,228,518	7,704,592	16,523,926	2,436,328	14,087,598

Input to, and output of, imputation using 1KG or TOPMed reference panels of total or genotyping-matched datasets. Imputed SNP numbers are after removing monomorphic, non-biallelic SNPs and SNPs with estimated imputation quality $\widehat{r^2}$ < 0.3. SNP, single nucleotide polymorphism; MAC, minor allele count; MAF, minor allele frequency

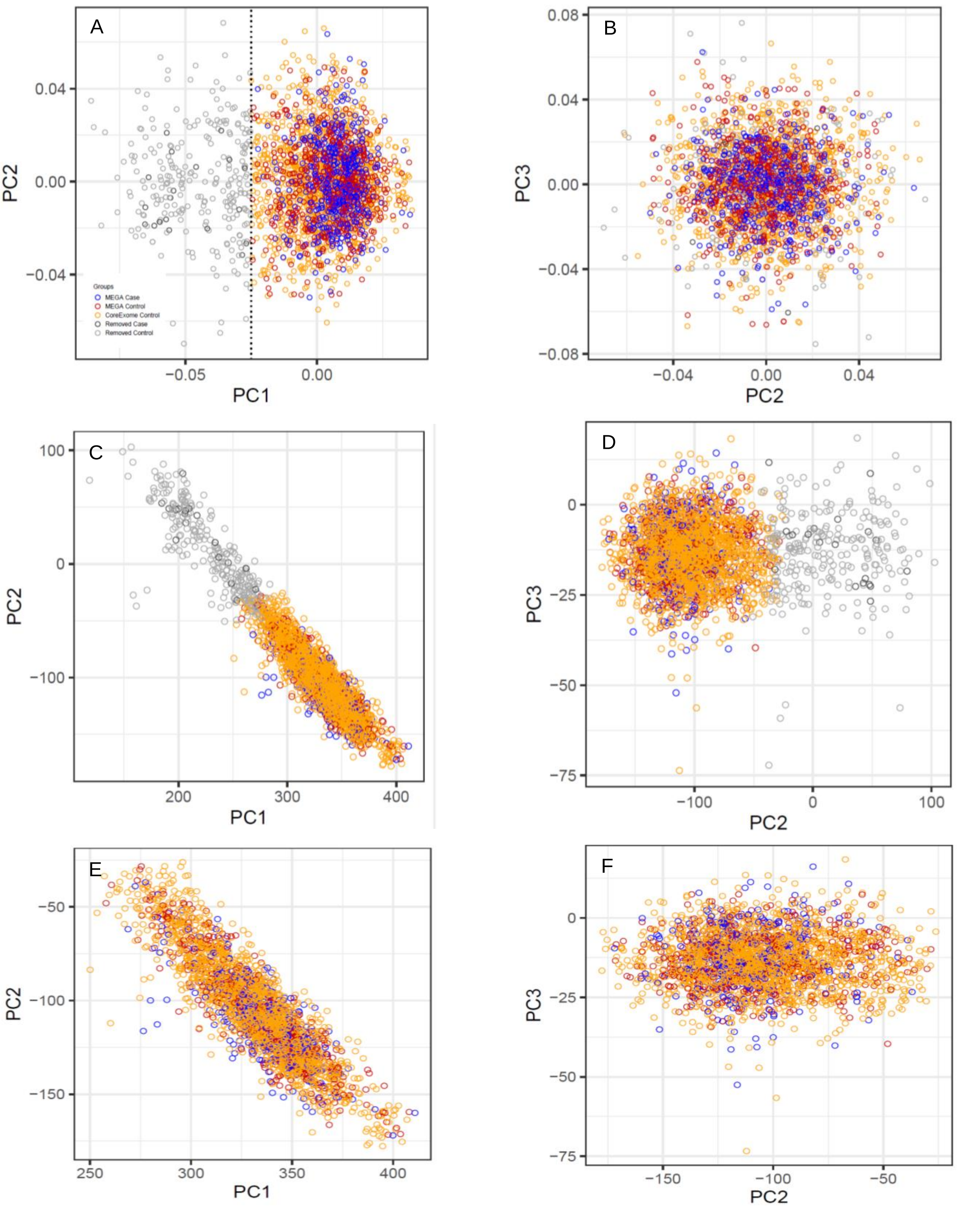

Figure S1. Genotype Principal Components Analysis (PCA) from PLINK (A-B) and Genetic Ancestry PCA from LASER/TRACE (B-D) of Total Sample before (A-D) and after (E-F) removing samples with PC1 < -0.025. Pneumonia samples denoted by case/control status, genotyping array, and if removed from further analysis. A, C and E, PC1 vs PC2; B, D and E, PC2 vs PC3; PC, principal component.

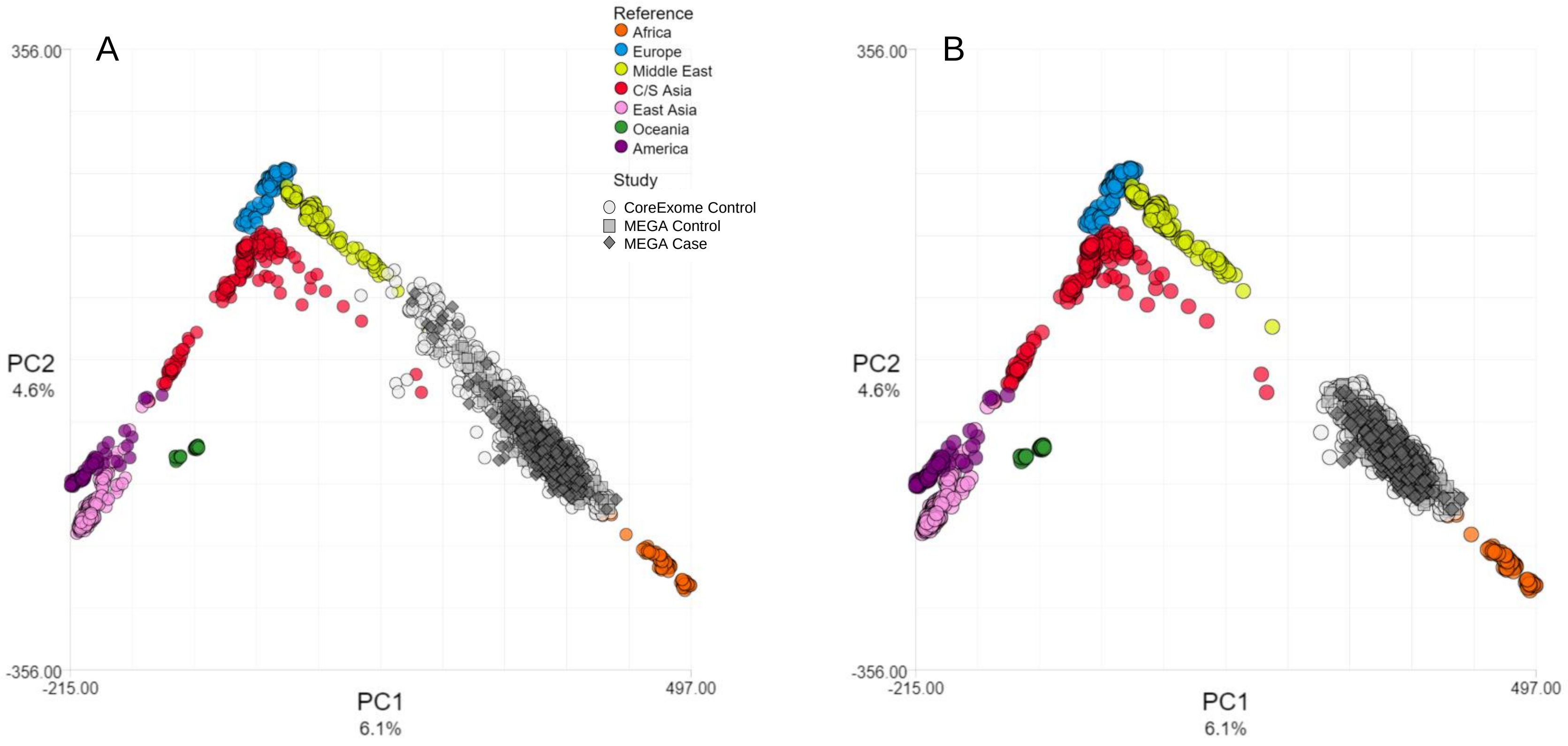

Figure S2. Genetic Ancestry Principal Components from LASER/TRACE Analysis in the Total Sample and Human Genome Diversity Project (HGDP) Samples. Pneumonia study samples denoted by case/control status and genotyping array. A, total sample prior to removing samples for PC1 < -0.025 in PLINK PCA; B, final total sample set. A and B, PC1 vs PC2; PC, principal component.

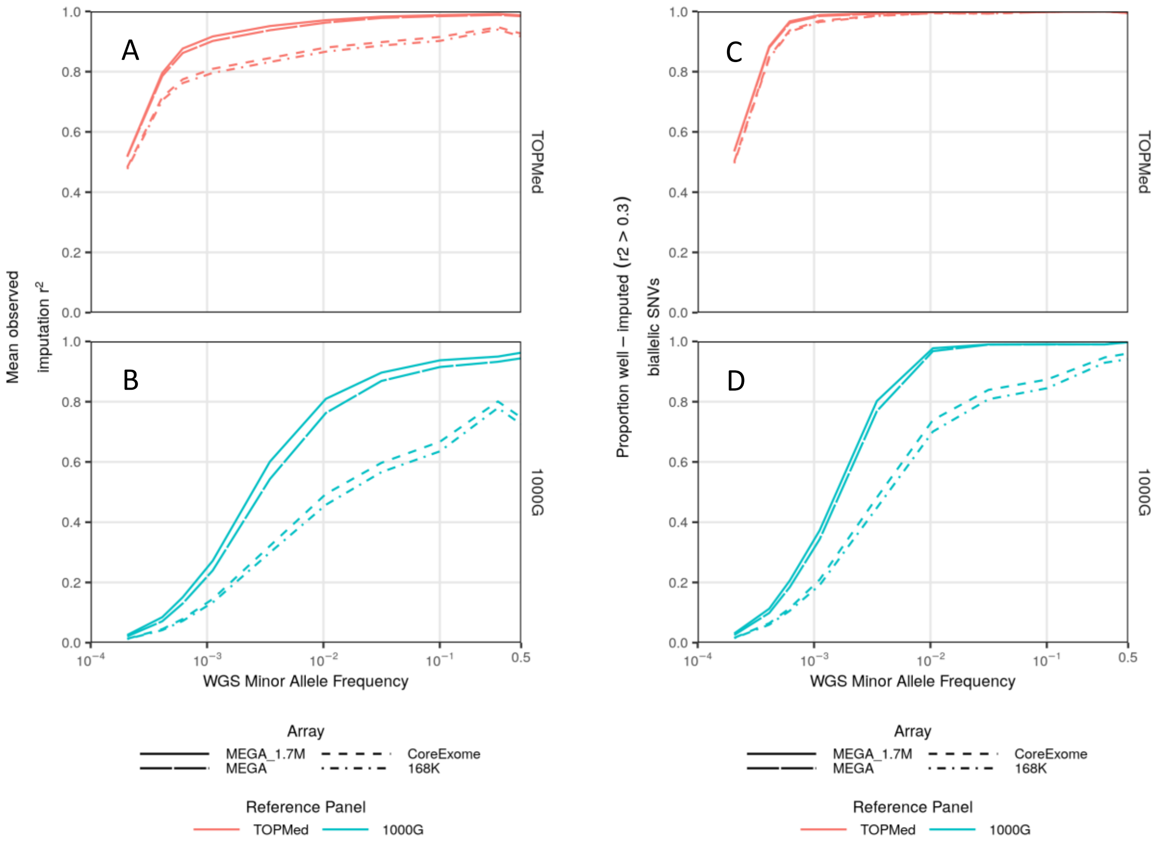

Figure S3. Observed Imputation Quality of InPSYght Genotypes Observed imputation correlation r^2 of whole genome sequencing (WGS) InPSYght genotypes with imputed genotype dosages from TOPMed (A,C) or 1KG (B, D) reference panels using chr11 SNPs. Array genotypes from MEGA (835K SNPs), total dataset (168K SNPs), and CoreExome array (188K SNPs). A & B, mean observed imputation r^2 for a given array vs InPSYght WGS minor allele frequency (MAF); C & D, proportion of imputed biallelic single nucleotide variants (SNVs) with observed $r^2 > 0.3$ by InPCYght WGS MAF.

Table S2. Average Effective Sample Size Adjusted for Imputation

Sample Name	Number of Variants in Imputation	Total Sample Size, N	Effective Sample Size, N_effective	Average Effective Sample Size Adjusted for Imputation, N_effective_imputation			
				MAF >0.01		rs334	
				1 KG	TOPMed	1 KG	TOPMed
Total	168,220	2530	1562	968	1406	1125	NA
Genotyping-matched	834,828	1000	999	879	979	749	NA

Effective samples size adjusted for mean observed chromosome 11 SNP imputation quality is (r^2) x (N_effective_imputation). Effective samples size adjusted for rs334 observed imputation quality is (r^2) x (N_effective_imputation_rs334). MAF, minor allele frequency

Table S3. Estimated and Observed Imputation Quality of β-globin SNPs Imputed with 1KG or TOPMed Reference Panels

SNP	hg38 Position	Estimated Imputation Quality ($\hat{r}^2$) for Indicated Samples, Genotyping Array Dataset and Reference Panel				Observed Imputation Quality (r^2) for Indicated Samples, Genotyping Array Dataset and Reference Panel			
		Total (168K SNPs)		Genotyping-matched subset (835K SNPs)		Total (168K SNPs)		Genotyping-matched subset (835K SNPs)	
		1KG	TOPMed	1KG	TOPMed	1KG	TOPMed	1KG	TOPMed
rs334	5227002	0.81	NA	0.92	NA	0.72	NA	0.75	NA
rs33930165	5227003	0.66	0.92	0.98	1.00	0.55	0.87	0.92	0.98
rs2226952	5285924	0.75	0.94	0.97	1.00	0.59	0.90	0.97	0.99

NA, not applicable as rs334 is not imputed in TOPMed; SNP, single nucleotide polymorphism

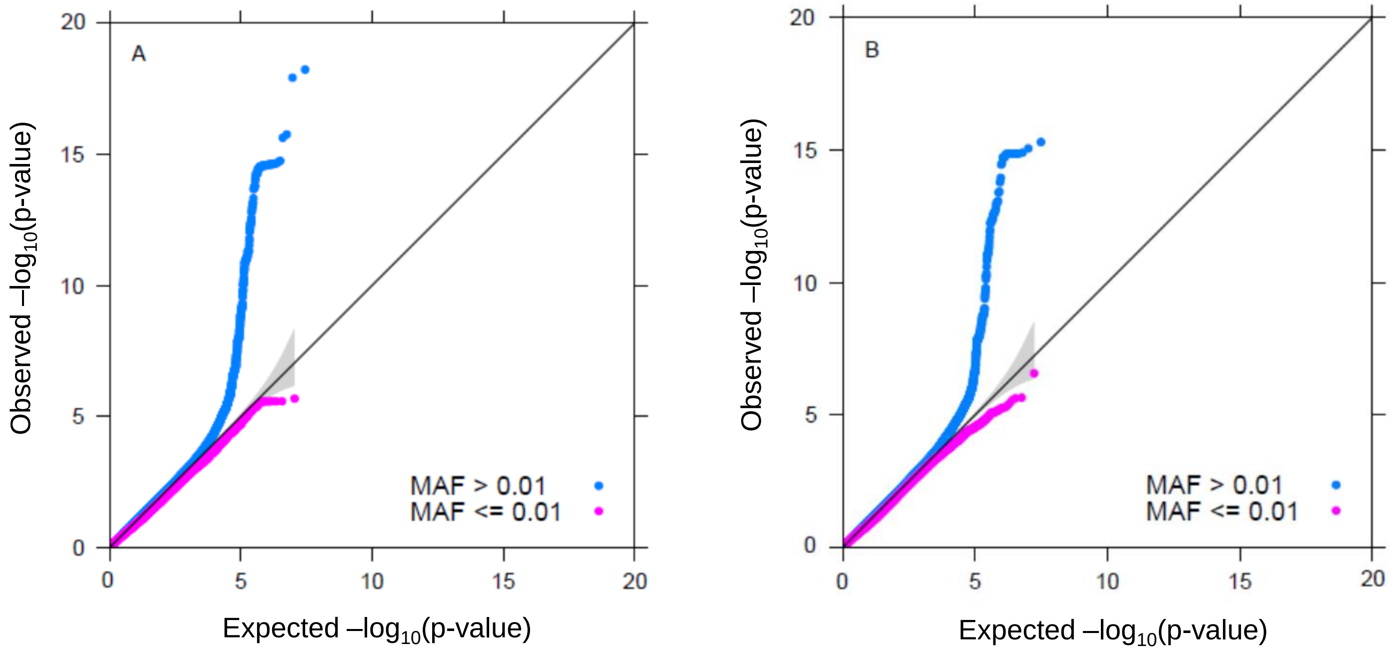

Figure S4. Total Sample Pneumonia GWAS QQ Plots QQ Plots of total sample pneumonia GWAS of single nucleotide polymorphisms (SNPs) imputed with 1KG (A) and TOPMed (B). Firth logistic regressions for SNPs with allele count ≥ 10. Plotted by minor allele frequency (MAF) bin. Solid line y=x.

Table S4. Total Sample β-globin Locus SNP Risk Allele Frequencies by Pneumonia Case/control Status and by Imputation Reference Panel

SNP	Risk/non-risk alleles	Position	Risk Allele Frequencies							
			1KG Reference Panel				TOPMed Reference Panel			
			Cases: MEGA	Controls: MEGA	Controls: CoreExome	Controls: MEGA & CoreExome	Cases: MEGA	Controls: MEGA	Controls: CoreExome	Controls: MEGA & CoreExome
rs334	A/T	5,227,002	0.16	0.059	0.063	0.062	NA	NA	NA	NA
rs33930165	T/C	5,227,003	0.033	0.013	0.012	0.012	0.035	0.0086	0.011	0.010
rs2226952	G/T	5,285,924	0.23	0.12	0.13	0.13	0.24	0.12	0.14	0.13

All samples were imputed with 168K array. The *HBB* gene is transcribed from the reverse strand of the genome (alleles called on the forward strand). Position, genome build GRCh38/hg38 base position on chromosome 11; SNP, single nucleotide polymorphism; NA, not applicable as rs334 is not imputed in TOPMed.

Table S5. Linkage Disequilibrium Between Selected SNPS in the Total Sample and Cases only

	Linkage disequilibrium in total sample/cases only (r^2 unshaded, D' shaded)					
	1KG			TOPMed		
	rs334	rs33930165	rs2226952	rs334*	rs33930165	rs2226952
rs334		0.01/0.03	0.27/0.33		0.01/0.03	0.19/0.25
rs33930165	0.24/0.42		0.06/0.11	0.17/0.42		0.06/0.07
rs2226952	0.64/0.62	0.92/1.0		0.64/0.63	0.83/0.82	

Linkage disequilibrium r^2 (unshaded) and D' (shaded) between rs334, rs33930165, and rs2226952 by imputation reference panel in the total sample/cases only. *LD to rs334 in TOPMed based on 1KG rs334 dosages

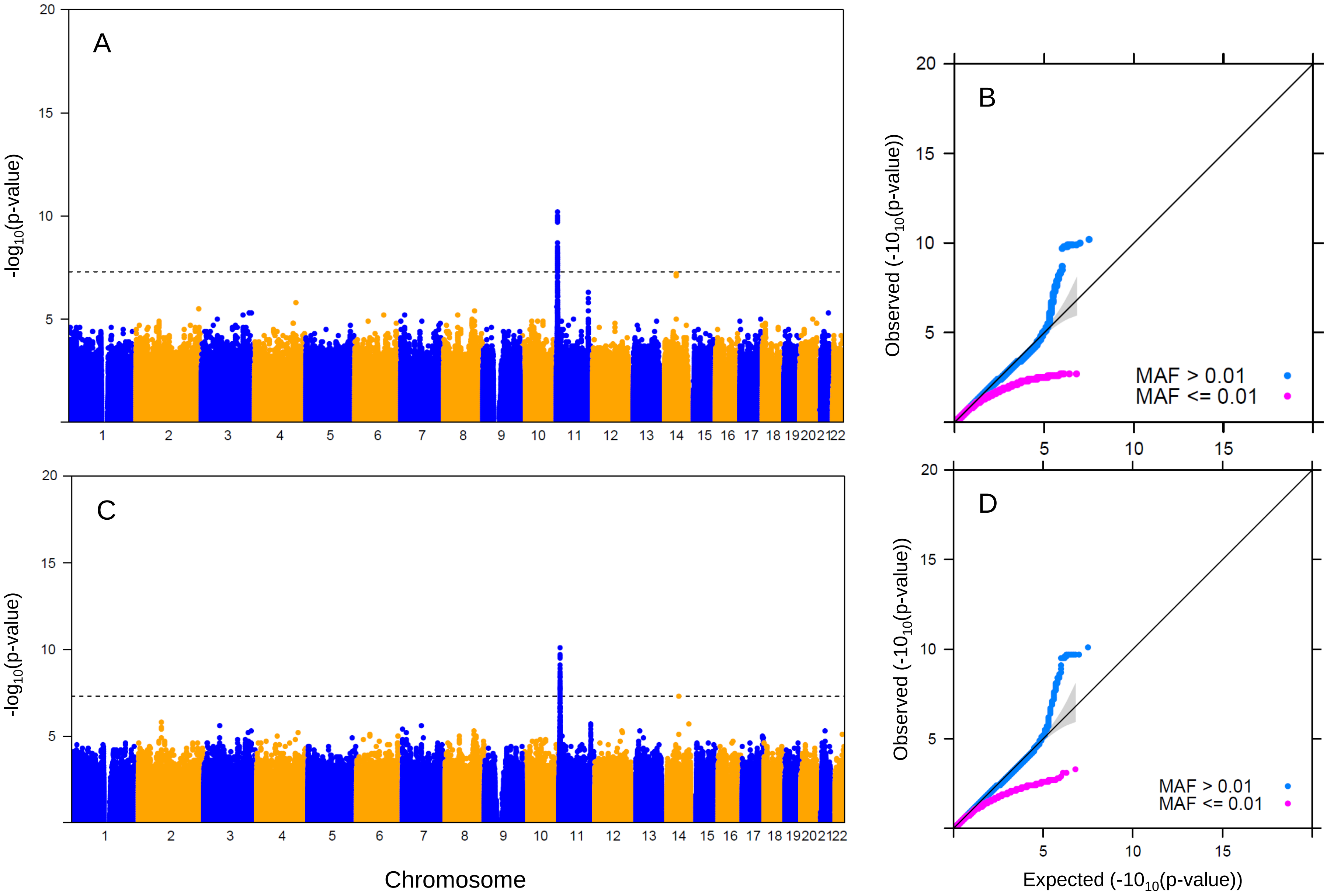

Figure S5. Genotyping-matched Subset Pneumonia GWAS Manhattan and QQ Plots by Imputation Reference Panel
Manhattan (A, C) and QQ plots (B, D) of genotyping-matched subset imputed with TOPMed (top) and 1KG (bottom). Firth logistic regressions of SNPs with allele counts ≥ 10. Dotted line, $p = 5 \times 10^{-08}$; solid line $y = x$.

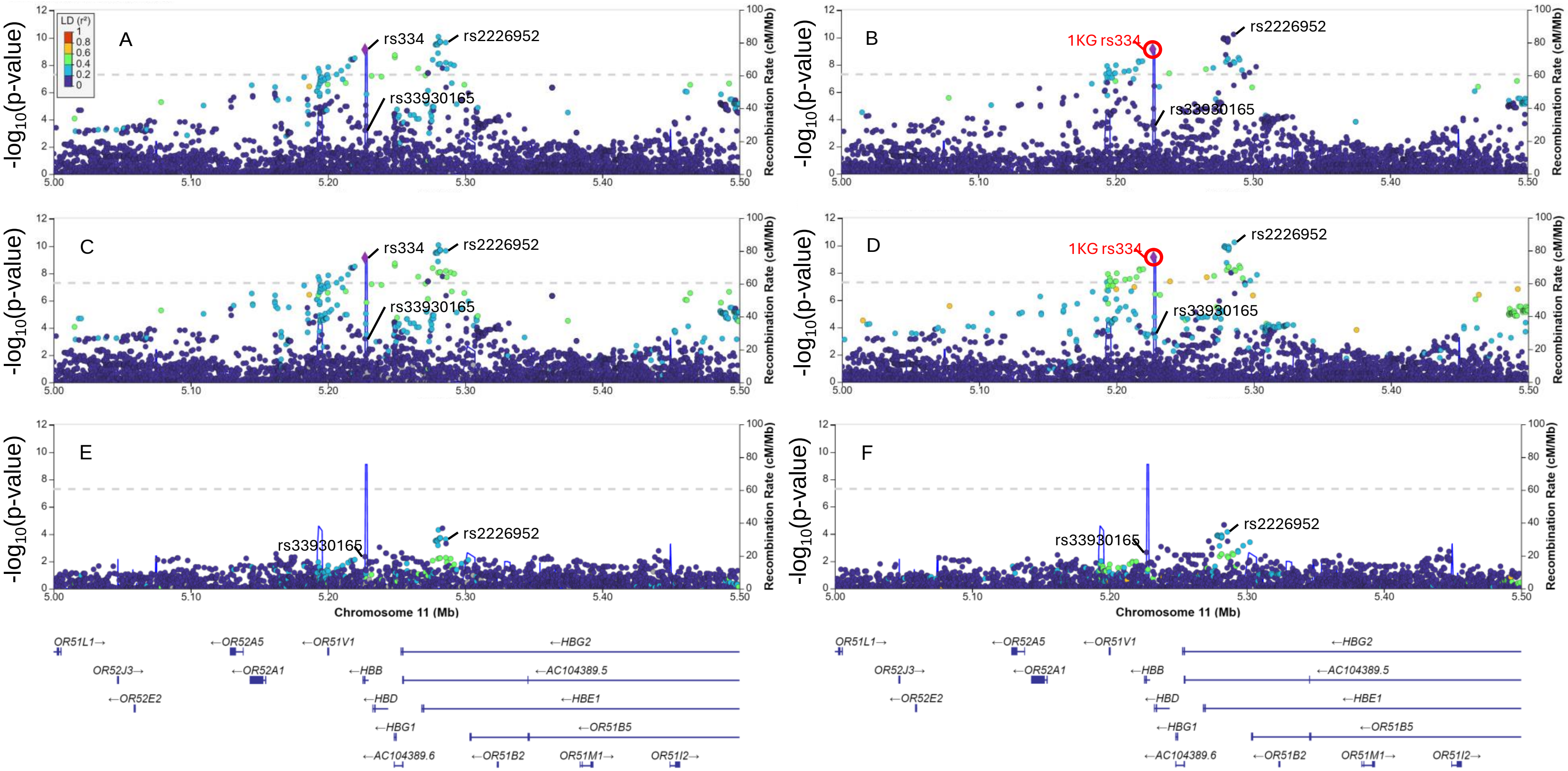

Figure S6. Genotyping-matched Subset β-globin Locus Plots of Pneumonia GWAS Results with and without Conditioning on 1KG rs334 dosages (colored by r^2 to 1KG imputed rs334) Plots of β-globin locus for the genotyping-matched subset with 1KG (left) and TOPMed (right) imputation. r^2 to rs334 (from 1KG imputation) in all samples (A, B), and in cases only (C-F). GWAS analysis conditioned on rs334 1KG dosages shown in E-F. Variant and gene positions shown in build hg38; 1KG rs334 added to TOPMed plots for comparison purposes; dotted line, 5×10^{-08}.

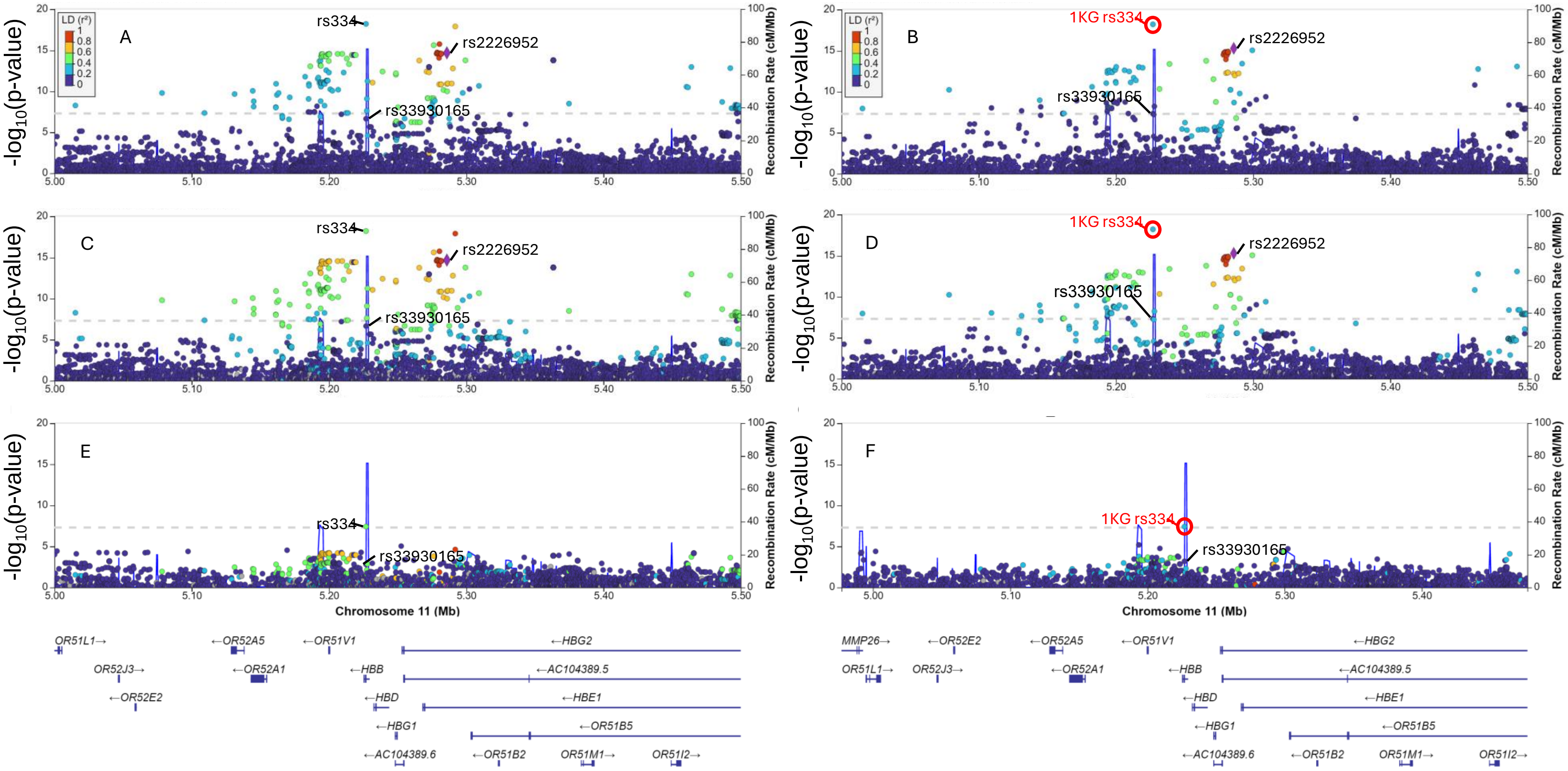

Figure S7. Total Sample β-globin Locus Plots of Pneumonia GWAS Results with and without Conditioning on rs2226952 Dosages (colored by r^2 to 1KG imputed rs2226952) Plots of β-globin locus for the genotyping-matched subset with 1KG (left) and TOPMed (right) imputation. r^2 to rs2226952 in all samples (A, B), and in cases only (C-F). GWAS analysis conditioned on rs2226952 dosages shown in E-F. Variant and gene positions shown in build hg38; 1KG rs334 added to TOPMed plots for comparison purposes; dotted line, 5×10^{-08}.

Table S6. Total Sample Pneumonia GWAS Results for Selected β-globin Locus SNPs with and without Conditioning on rs2226952

SNP	Risk/Non-risk Alleles	1KG				TOPMed			
		Unconditioned		Conditioned on rs2226952		Unconditioned		Conditioned on rs2226952	
		OR (95% CI)	p-value	OR (95% CI)	p-value	OR (95% CI)	p-value	OR (95% CI)	p-value
rs334	A/T	2.76 (2.21-3.46)	5.9 x 10^{-19}	2.10 (1.62-2.72)	3.8 x 10^{-08}	NA	NA	2.09[a] (1.61-2.70)	1.9 x 10^{-08}
rs33930165	T/C	4.44 (2.52-7.82)	2.2 x 10^{-07}	2.59 (1.45-4.65)	1.5 x 10^{-03}	3.89 (2.38-6.35)	4.7 x 10^{-08}	2.42 (1.46-4.01)	6.5 x 10^{-04}
rs2226952	G/T	2.29 (1.86-2.81)	2.0 x 10^{-15}	--	--	2.14 (1.78-2.57)	5.1 x 10^{-16}	--	--

Total dataset imputed with 1KG or TOPMed reference panels and analyzed using Firth logistic regression with and without conditioning on rs2226952 dosage. The *HBB* gene is transcribed from the reverse strand of the genome (alleles called on the forward strand). [a]1 KG rs334 OR, 95% CI, and p-value after conditioning with TOPMed rs2226952 gene dosages; SNP, single nucleotide polymorphism; OR, odds ratio; CI, confidence interval; NA, not applicable as rs334 is not imputed in TOPMed

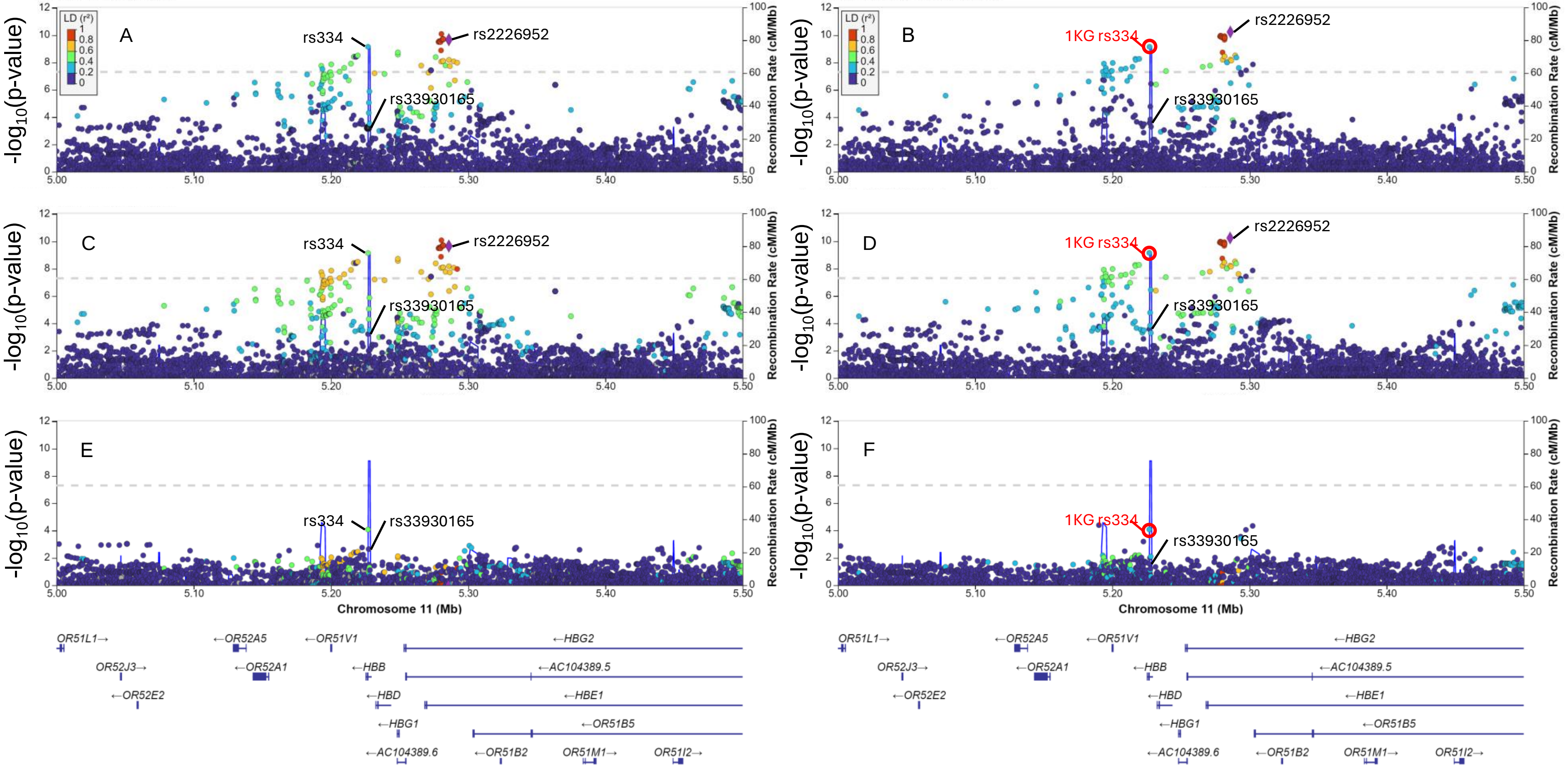

Figure S8. Genotyping-matched Subset β-globin Locus Plots of Pneumonia GWAS Results with and without Conditioning on 1KG rs2226952 Dosages (colored by r^2 to 1KG imputed rs2226952) Plots of β-globin locus for the genotyping-matched subset with 1KG (left) and TOPMed (right) imputation. r^2 to rs2226952 in all samples (A-B), and in cases only (C-F). GWAS analysis conditioned on rs2226952 dosages shown in E-F. Variant and gene positions shown in build hg38; 1KG rs334 added to TOPMed plots for comparison purposes; dotted line, 5×10^{-08}.

Table S7. Genotyping-matched Subset Pneumonia GWAS Results for Selected β-globin SNPs with and without Conditioning with rs334 or rs2226952

Imputation Reference Panel	SNP	Risk/Non-risk alleles	Unconditioned		Conditioned on rs334		Conditioned on rs2226952	
			OR (95% CI)	p-value	OR (95% CI)	p-value	OR (95% CI)	p-value
1 KG	rs334	A/T	2.72 (1.98-3.75)	7.1×10^{-10}	--	--	2.03 (1.42-2.90)	8.4×10^{-05}
	rs33930165	T/C	3.26 (1.65-6.42)	6.1×10^{-04}	2.72 (1.36-5.44)	4.5×10^{-03}	1.98 (0.98-4.02)	5.7×10^{-02}
	rs2226952	G/T	2.18 (1.71-2.78)	2.2×10^{-10}	1.67 (1.27-2.20)	2.4×10^{-04}	--	--
TOPMed	rs334	A/T	NA	NA	NA	NA	NA	NA
	rs33930165	T/C	3.73 (1.83-7.61)	2.7×10^{-04}	3.08 (1.49-6.36)	2.24×10^{-03}	2.29 (1.09-4.79)	2.7×10^{-02}
	rs2226952	G/T	2.22 (1.75-2.82)	5.8×10^{-11}	1.72 (1.32-2.26)	7.15×10^{-05}	--	--

Genotype-match subset imputed using 1KG or TOPMED reference panels and analyzed using Firth logistic regression without and with conditioning on 1KG-imputed rs334 or rs2226952 (from respective imputations). The *HBB* gene is transcribed from the reverse strand of the genome (alleles called on the forward strand). SNP, single nucleotide polymorphism, OR, odds ratio; CI, confidence interval; NA, not applicable as rs334 in not imputed with TOPMed.

Table S8. TOPMed imputed Total Sample Pneumonia GWAS Genome-wide Significant SNP after Conditioning with 1KG rs334 Dosages

SNP	Risk/Non-risk alleles	1KG					TOPMed				
		Unconditioned		Conditioned on rs334		$\widehat{r^2}$	Unconditioned		Conditioned on rs334		$\widehat{r^2}$
		OR (95% CI)	p-value	OR (95% CI)	p-value		OR (95% CI)	p-value	OR (95% CI)	p-value	
rs12137603	A/C	1.49 (1.06 - 2.09)	0.020	1.48 (1.05 - 2.09)	0.023	0.42	1.81 (1.45 - 2.25)	1.6×10^{-07}	1.88 (1.50 - 2.35)	4.2×10^{-08}	0.86

Total sample imputed with 1 KG TOPMed reference panel Firth logistic regression result upon conditioning with 1KG rs334 dosages. rs12137603 in genome build GRCh38/hg38 at position 240,484,996 in chromosome 1. SNP, single nucleotide polymorphism; $\hat{r}^2$, estimated imputation quality for given imputation; OR, odds ratio; CI, confidence interval.

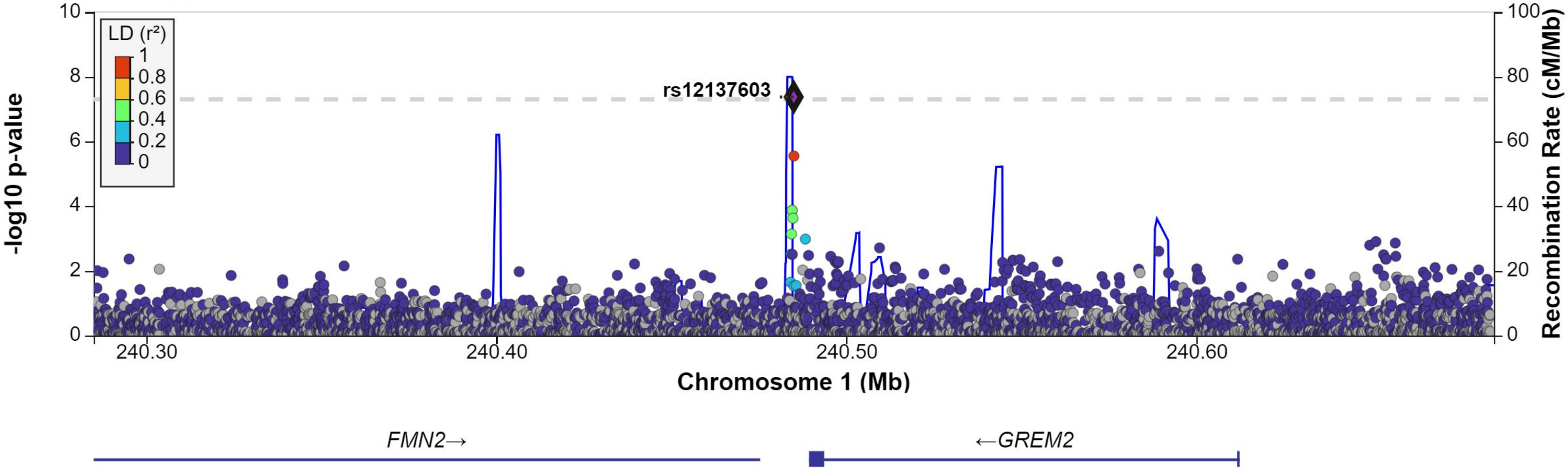

Figure 9S. Total Sample Locus Plot of GWAS Genome-wide Significant SNP after Conditioning on 1KG rs334 Dosages. TOPMed imputation with LD to rs12137603 from African population in 1KG reference panel. Variant and gene positions shown in build hg38, dotted line, 5×10^{-08}.

Table S9. Pneumonia GWAS Results for Selected β-globin SNPs with and without Individuals with Sickle Cell Disease

Imputation Reference Panel	SNP	Risk/Non-risk alleles	Total Sample		Without Individuals with SCD	
			OR (95% CI)	p-value	OR (95% CI)	p-value
1 KG	rs334	A/T	2.76 (2.21-3.46)	5.9×10^{-19}	1.24 (0.88-1.76)	0.22
	rs33930165	T/C	4.44 (2.52-7.82)	2.2×10^{-07}	3.08 (1.61-5.90)	6.2×10^{-04}
	rs2226952	G/T	2.29 (1.86-2.81)	2.0×10^{-15}	1.49 (1.17-1.90)	1.1×10^{-03}
TOPMed	rs334	A/T	NA	NA	NA	NA
	rs33930165	T/C	3.89 (2.38-6.35)	4.7×10^{-08}	2.78 (1.58-4.85)	3.2×10^{-04}
	rs2226952	G/T	2.14 (1.78-2.57)	5.1×10^{-16}	1.54 (1.25-1.91)	5.0×10^{-05}

Total sample imputed using 1KG or TOPMED reference panels. Total sample with and without individuals with SCD analyzed using Firth logistic regression. The *HBB* gene is transcribed from the reverse strand of the genome (alleles called on the forward strand). SNP, single nucleotide polymorphism, SCD, sickle cell disease; OR, odds ratio; CI, confidence interval; NA, not applicable as rs334 in not imputed with TOPMed
